# Avoidance, confusion or solitude? Modelling how noise pollution affects whale migration

**DOI:** 10.1186/s40462-024-00458-w

**Published:** 2024-02-19

**Authors:** Stuart T. Johnston, Kevin J. Painter

**Affiliations:** 1 School of Mathematics and Statistics, The University of Melbourne, Parkville, VIC 3010 Australia; 2 Dipartimento Interateneo di Scienze, Progetto e Politiche del Territorio (DIST), Politecnico di Torino, 39, 10125 Turin, Italy

**Keywords:** Collective navigation, Baleen whales, Acoustic communication, Noise pollution

## Abstract

Many baleen whales are renowned for their acoustic communication. Under pristine conditions, this communication can plausibly occur across hundreds of kilometres. Frequent vocalisations may allow a dispersed migrating group to maintain contact, and therefore benefit from improved navigation via the “wisdom of the crowd”. Human activities have considerably inflated ocean noise levels. Here we develop a data-driven mathematical model to investigate how ambient noise levels may inhibit whale migration. Mathematical models allow us to simultaneously simulate collective whale migration behaviour, auditory cue detection, and noise propagation. Rising ambient noise levels are hypothesised to influence navigation through three mechanisms: (i) diminished communication space; (ii) reduced ability to hear external sound cues and; (iii) triggering noise avoidance behaviour. Comparing pristine and current soundscapes, we observe navigation impairment that ranges from mild (increased journey time) to extreme (failed navigation). Notably, the three mechanisms induce qualitatively different impacts on migration behaviour. We demonstrate the model’s potential predictive power, exploring the extent to which migration may be altered under future shipping and construction scenarios.

**Supplementary Information:**

The online version contains supplementary material available at 10.1186/s40462-024-00458-w.

## Introduction

Many baleen whales routinely perform immense migrations [1], with individual whales observed travelling close to 20,000 km in a single year [2]. This clearly represents a significant investment of time and energy. The inherent difficulties of observing whale behaviour leaves numerous questions about navigation unanswered, not least the nature of navigation cues [1, 3]. One factor that has received considerable attention, though, is the whales’ ability to detect, respond to, and produce sounds [4]. Notably, sound propagates rapidly in water with little transmission loss, allowing information to be signalled/received across large distances [5]. External sound sources may provide navigating cues [3, 6], while emitting low frequency sounds may provide information about the bathymetric features of the environment [7].

Studies into “whalesong” that date back over half a century [8] have increased awareness of acoustic whale communication. In the context of navigation, this has been suggested to allow whales to broadcast and reinforce route information [5]. Such collective navigation has been investigated both experimentally and through modelling [9]. In the latter, collective behaviour has been shown to improve migration efficiency through a “many wrongs” principle [10] that reduces individual-level uncertainty, particularly if intrinsic navigation information is low [11, 12]. The many wrongs principle suggests that collective navigation is more effective due to the averaging out of navigational errors across the group [11]. Baleen whales can generate loud and low frequency sounds that lead to extraordinary communication ranges, theoretically covering hundreds of kilometres [4, 5]. As such, seemingly-isolated whales may still be benefiting from collective navigation via long-distance communication across a widely dispersed group [5, 13]. Substance to such conjectures can be found in the sequences of calls made by humpbacks, tens of kilometres apart, while traversing migration routes [14] or the apparent exchange of calls between bowhead whales as they navigate around ice [15].

**Fig. 1:**
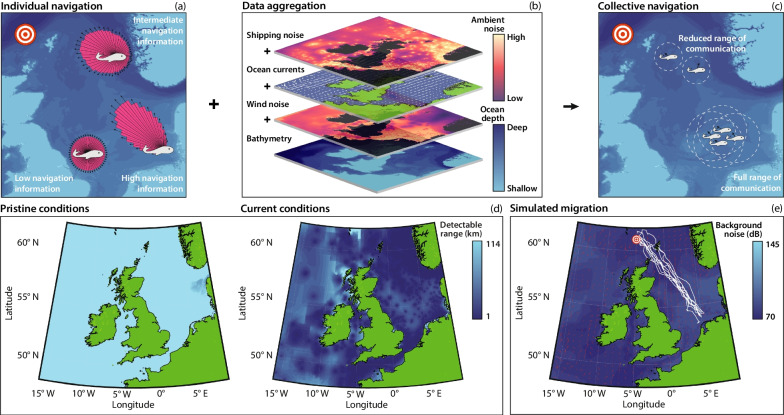
Data-driven agent-based modelling framework. **a**–**c** Schematic of the data-driven agent-based model of whale migration. **a** Individual navigation relies on inherent navigation that may be impacted by local factors. **b** Data sources incorporated in the model. **c** Collective navigation relies on the detection of signals from other whales, which is influenced by the local ambient noise. **d** The detectable range of whale calls for our model in a pristine soundscape (wind noise only) and the current soundscape (wind and shipping noise). **e** Representative, randomly-selected, trajectories from the model of collective navigation in a pristine soundscape

The distance that a sound remains detectable in the ocean depends on fixed elements, such as bathymetry, and changing elements, such as ambient noise [16]. In the pre-industrial ocean, ambient noise would have been generated from factors such as wind, rain, breaking ice and biotic sources. Today, ambient noise in the ocean is increasingly a result of human activities [16, 17]. Anthropogenic sound sources include those from shipping, sonar, exploration, and offshore construction; estimates suggest that these may have already induced a rise of more than 20 dB in certain regions [16]. Given the importance of sound for communication and information, this noise pollution is believed to have a broad spectrum of impacts on whales (and other marine animals) [17–19]. This ranges from a reduction in communication range [5, 20] to physiological damage and stranding events that follow extreme noise events [19]. Observed reactions include altered swimming behaviour due to noise avoidance responses [21–23], and increased call volumes (Lombard effect) under higher ambient noise levels [24, 25]. Independent of any direct behavioural change, the communication range is still significantly diminished [24–26].

Mathematical models provide a framework to investigate the interplay between cue detection, noise pollution, and navigation behaviour. Abstract models have previously been developed to explore the benefit of collective navigation [9, 11, 12, 27, 28]. Broadly, we observe an increase in navigational efficiency with an increase in the number of observable conspecifics within a group; with specific investigations that include, for example, “leaders” in the population [29], individual heterogeneity [30], or flowing environments [31]. We refer the interested reader to the review by Berdahl et al. for a more detailed summary [9]. Whale-specific models have been presented to investigate, for example, the migration of humpback whales [32], and the migration and foraging behaviour of blue whales [33]. However, while certain realistic aspects of whale migration have been included in these models, the importance of collective navigation in such models remains to be explored, despite its conjectured importance [5, 13, 14]. In particular, the presence of a spatially- and temporally-varying noise field and its interaction with the communication range of a whale population has yet to be incorporated in a mathematical model of collective navigation and migration.

The aim of this study is to assess the extent to which ambient noise can influence whale migration paths. The lack of data under controlled conditions (as noted “baleen whales are reticent laboratory subjects” [5]) and the infeasibility of reverting from the current soundscape to a pristine soundscape motivates our computational modelling approach. Here we explore how anthropogenic activity may negatively impinge on the navigating ability of whales. Specifically, we generate synthetic migration paths while systematically addressing three possible consequences of higher noise: reduced communication space (‘solitude’), reduced goal-targeting information (‘confusion’), and the triggering of explicit reorientation responses (‘avoidance’). These plausible repercussions of increased noise levels are built into an agent-based mathematical model for whale movement that incorporates multiple layers of environmental data.

## Results

Our mathematical model is designed to simulate a virtual population of baleen whales as they migrate across the North Sea; for example, a population returning from a feeding area. While the model does not explicitly describe a particular whale species, we have parameterised the model as much as possible from data of minke whales (*Balaenoptera acutorostrata*); we note that the model could be parameterised via other whale species and other migration routes, given suitable data. The agent-based model builds on a collective navigation model introduced in [12], which assumes that the confidence in the target direction of each whale combines local inherent information, e.g. navigation cues, (Fig. 1a) and the collective information gained by co-aligning movement paths with other whales in communication range (Fig. 1c).

In the model, the communication range is principally dictated by the ocean ambient noise (Fig. 1d); other factors include the sound transmission decay and source level. We decompose the ambient noise into surface wind and shipping noise levels, which allow us to consider two forms of ocean soundscape: the *pristine* soundscape (wind noise only) and the *current* soundscape (wind and shipping noise) [34]. These noise layers represent two of the four data sources needed for model implementation (Fig. 1b); the other data are ocean currents [35] and bathymetry [36]. A detailed explanation of the model and the parameters used, and a sensitivity analysis can be found in the “Methods” section and the Supplementary Information,  which includes an Overview, design concepts and details protocol (Additional file 1) [37, 38]. The model relies on noise response mechanisms that are, by necessity, speculative due to our current understanding of baleen whales. The conclusions arising from our model should therefore be interpreted with this in mind. However, the clear qualitative differences in migration patterns that emerge from our model imply that it may be possible to identify the dominant type of response to noise from observational data in future.

### Migration in the pristine soundscape

**Fig. 2:**
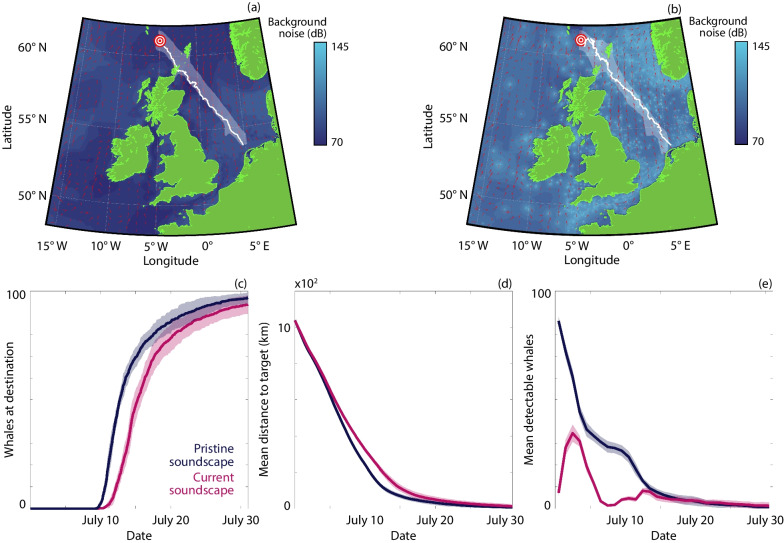
Comparison of navigation in the pristine and current soundscapes. **a** Median and spread of whale trajectories in the pristine soundscape. **b** Median and spread of whale trajectories in the current soundscape. **c** The number of whales that have arrived at the target destination. **d** The mean distance of the population from the target. **e** The mean number of detectable whales (averaged daily). Results are presented for the pristine (dark blue) and current (magenta) soundscapes. The lines and ribbons correspond to the mean ± one standard deviation over 10 simulations

The pristine soundscape serves as the benchmark, representing navigation in a pre-industrial ocean and offering optimal conditions for navigation: communication is close to maximum across the migration route (Fig. 1d). Representative migration trajectories show broadly straight line movements towards the target (Fig. 1e). At a population level, trajectories are constrained to a relatively tight corridor (Fig. 2a), implying that a high degree of cohesion is maintained throughout migration, despite the lack of an explicit “attraction” mechanism between whales in the model. However, this does not imply physical proximity: average pairwise distance is $\sim 100$ km and only $\sim 5\%$ of the group lie within 5 km of each other, so visual sightings of pairs or groups would form relatively rare events. The mean distance to the destination decreases linearly (Fig. 2d), with the majority of whales arriving within a few days of each other (Fig. 2c). The tail is attributed to a few “straggling” outliers, such as those either positioned at the group edge or adopting routes that require navigation about obstacles such as islands. The number of detected whales indicates the level of communication. This is initially high while whales are co-located at the feeding ground, but drops with migration as the group becomes dispersed. Crucially, the number of detectable whales remains high enough to benefit navigation. We do not speculate on the rate of calls for individual whales. Rather, we assume that a whale calls more often than it undergoes reorientation so that contemporaneous information is available; for example, calling rates of individual North Atlantic minke whales vary between 8.7 and 133.3 calls/hr, with a median intercall interval of 1 min [39]. Note that during the final stages, the bathymetry helps to funnel the population between landmasses, illustrating a geophysical influence on group structure and navigation; here, land avoidance behaviour can dominate by orienting individuals away from excessively shallow waters to prevent beaching (Additional file 2: Fig. S3).

### Reduced communication range delays arrival

We now consider the current soundscape, which includes shipping noise (Fig. 2b) [34]. The increase in ambient noise slows migration, introducing a delay via a 3–4 day shift in the distribution of arrival times (Fig. 2c). This effectively represents an additional $\sim 20\%$ in travel time. Longer migration primarily results from reduced communication space, as the detectable range drops by an order of magnitude or so across the migration (Fig. 1d). This manifests in a dramatic decrease in the number of detectable calls (Fig. 2e), when compared against the pristine soundscape. This is particular apparent from the outset, where initial proximity to shipping lanes leads to considerably reduced communication. Calling recovers as the population moves into quieter seas. The funneling between landmasses towards the end of the migration proves particularly beneficial here, bringing individuals within communication range despite the noise-induced masking. Overall, though, throughout migration the communication range is greatly diminished and the advantages of collective navigation are lost, with individuals more heavily reliant on inherent information such as memory or cue detection. We see similar trends for different choices of the relative weight placed on inherent and collective information (Additional file 2: Fig. S10).

### Noise avoidance can block migration routes

**Fig. 3:**
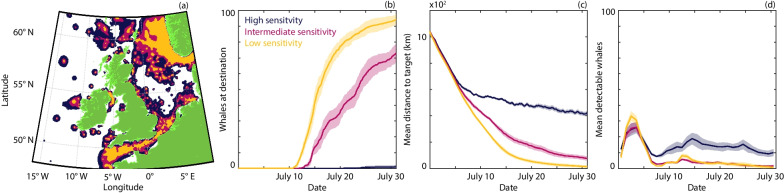
Comparison of navigation with different sensitivities to noise that induce an avoidance response. **a** Regions of the ocean that induce a noise avoidance response under low (orange), intermediate (orange+red) and high (orange+red+dark blue) sensitivities. **b** The number of whales that have arrived at the target destination. **c** The mean distance of the population from the target. **d** The mean number of detectable whales (averaged daily). Results are presented for high (dark blue), intermediate (magenta) and low (orange) sensitivities. The lines and ribbons correspond to the mean ± one standard deviation over 10 simulations

Explicit noise avoidance behaviour is included in Fig. 2, but is only triggered at higher exposure levels. Entirely eliminating this avoidance only marginally improves navigation (Additional file 2: Fig. S1), indicating that the impaired navigation observed in Fig. 2 primarily stems from communication masking. Clear noise avoidance responses are well documented for whales exposed to nearby loud noise sources and are typically based on direct visual observations [23]. As such, uncertainty exists regarding the level of noise at which such a response occurs, with more subtle path deviations difficult to assess visually. To explore the impact of explicit noise avoidance we lower the intensity threshold at which this behaviour is triggered (Fig. 3) (changing from $\sim$1 km to $\sim$8 km from a large ship). Navigation efficiency is significantly reduced, with a slower approach to the target and delayed arrival times are observed (Fig. 3b, c). Notably, significant differences only emerge around halfway through the journey. To understand this we chart the regions where noise avoidance is triggered for different sensitivities (Fig. 3a). The first portion of the migration path remains relatively clear for all sensitivities, yet later stage migration becomes more convoluted due to higher ambient noise. Consequently, routes that do not require noise avoidance become restricted to certain channels, which can be closed off as the sensitivity increases. This, in turn, leads to failures in migration (Fig. 3b–d), as individuals become trapped behind a wall of noise. We further explore this phenomenon by systematically varying the parameters in the noise avoidance mechanism (Additional file 2: Fig. S8–S9).

### Noise-induced reductions in inherent information

**Fig. 4:**
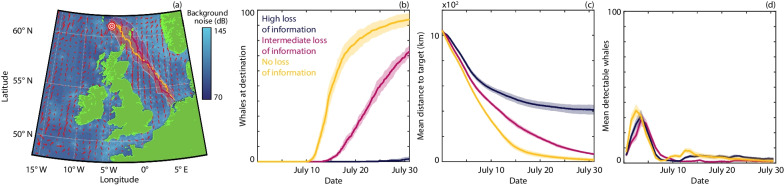
Comparison of different levels of loss of inherent information due to noise pollution. **a** Median and spread of whale trajectories. **b** The number of whales that have arrived at the target destination. **c** The mean distance of the population from the target. **d** The mean number of detectable whales (averaged daily). Results are presented for high (dark blue), intermediate (magenta) and no (orange) loss of inherent information. The lines and ribbons correspond to the mean ± one standard deviation over 10 simulations

Higher ambient noise may also reduce the level of inherent information available. This may occur either directly, through obscuring sound sources that serve as navigation cues; or indirectly, through poor processing of information due to noise-induced confusion or stress. Notably, we observe significant delays in arrival time when noise-induced information loss is included, becoming severe if the loss is high (Fig. 4). Crucially, this ineffective navigation does not result from closed routes but from less-directed movement that places whales at greater susceptibility to dispersing effects, including currents. This is particularly prominent across the second half of the migration, where the higher noise within this region results in greater spread and significant deviation of the median trajectory (Fig. 4a). Previous studies indicate that poor information zones can be compensated for through collective navigation [12], via a communication relay between information-rich and information-poor regions. Here, this compensation is unavailable as noise concurrently reduces group communication. Hence, in the model, an increase in ambient noise can have a compounding and negative impact on navigation efficiency due to simultaneously affecting the different elements that aid navigation.

**Fig. 5:**
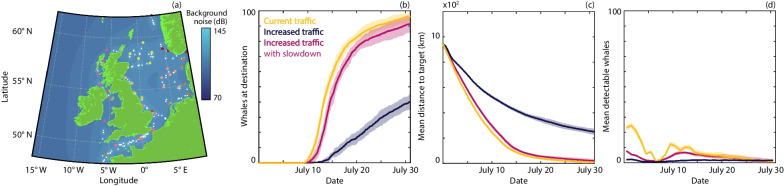
Comparison of navigation under different levels of shipping activity. **a** Representative noise map for shipping traffic. Start points, end points and waypoints for individual routes are shown in unique colours; ship locations are white. **b** The number of whales that have arrived at the target destination. **c** The mean distance of the population from the target. **d** The mean number of detectable whales (averaged daily). Results are presented for current shipping traffic (orange), increased traffic (dark blue) and increased traffic alongside noise mitigation measures (magenta). The lines and ribbons correspond to the mean ± one standard deviation over 10 simulations

**Fig. 6:**
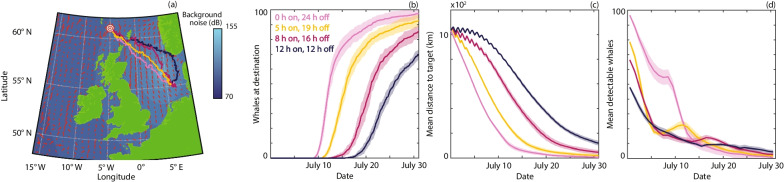
Comparison of navigation under different levels of construction activity in an otherwise pristine soundscape. **a** Noise map during active construction at 56.35 ^∘^N, 4.25 ^∘^E with median trajectories. **b** The number of whales that have arrived at the target destination. **c** The mean distance of the population from the target. **d** The mean number of detectable whales (averaged daily). Results are presented for 0 h (pink), 5 h (orange), 8 h (magenta) or 12 h (dark blue) of construction activity per day. The lines and ribbons correspond to the mean ± one standard deviation over 10 simulations

### Perturbing the current soundscape

Building on the insights of the above analysis, the predictive potential of the model is demonstrated through perturbing the current soundscape (Fig. 5). Specifically, we (i) construct synthetic noise maps with shipping noise that originates from virtual vessels (Fig. 5a), and; (ii) consider the localised impact of a large scale offshore construction process in an otherwise pristine soundscape (Fig. 6). For the synthetic equivalent to the current soundscape we set parameters for the routes, numbers and source levels of virtual vessels to near recreate the migration behaviour within the current soundscape profile (Fig. 2). From this baseline we consider the impact of a future 50% increase in traffic (Fig. 5, dark blue), which has been suggested will occur before 2050 [40]. The resulting rise in ambient noise hinders migration through triggering the noise avoidance that traps a subset of the population behind high noise regimes. As a potential mitigation we explore the extent to which introducing slowdown zones can offset the impaired migration; trials indicate that slower speeds can reduce noise levels from certain vessels upwards of 10 dB [41]. Introducing a slowdown can partially recover migration efficiency (Fig. 5, magenta), despite the elevated vessel numbers. To simulate the impact of a construction project, we place a single noise source at a fixed location and consider different amounts of construction activity (e.g. pile driving) per day. Increasing construction activity leads to an increasing perturbation to the migration path (Fig. 6a) and day-to-day activity triggers oscillations in the remaining migration distance (Fig. 6c). The latter results from a daily triggering of noise avoidance behaviour that operates until the whales have moved sufficiently far away from the source to allow normal migration. The total amount of construction activity has a more pronounced impact on migration than the scheduling of the activity (Additional file 2: Fig. S5).

## Discussion

We have developed a model to explore the impact of ocean noise on whale migration routes, where higher ambient noise (i) reduces whale communication space, (ii) generates an avoidance response when sufficiently loud, and (iii) lowers inherent navigation information. Each mechanism can lengthen the journey time, and certain scenarios may even lead to failed migration. As such, the energetic cost of migrating in the current ocean soundscape is expected to be higher than in a pristine soundscape. Notably, though, each mechanism has a subtly different impact: it is not simply three different forms of slower migration. Rather, diminished communication space leads to greater solitude and slower migration as collective navigation benefits are eroded. Under a loss of information there is increased confusion, leading to off-course drifting and greater susceptibility to ocean currents. Finally, loud noises lead to a strong noise avoidance response and routes that become blocked, and hence migration may fail. Whether these distinct forms of trajectory perturbation predicted by our virtual whale model are also observed within tracked whales would be of key interest. The conclusions drawn from the model predictions are contingent on the relevance and accuracy of the mechanisms in the model. It is likely that baleen whales exhibit more sophisticated behaviour than that encoded into the model, including learning-type responses and adaptation to the evolving environmental conditions. However, in the absence of detailed understanding we sought to impose relatively simple mechanisms and explore how these mechanisms may manifest in qualitatively different migration behaviour.

Reduced communication spaces lengthen journey times through lower collective navigation. Previous theoretical studies [11, 12] indicate that co-alignment of migration paths provides a “many wrongs” [10] benefit by reducing inherent uncertainty, but only above a critical number of detectable neighbours [11, 12]. A reduced communication space can therefore eliminate this benefit, leading to a more convoluted path. We have not (explicitly) included noise compensation behaviour, such as the Lombard effect. This effect has been observed in whale populations, where call intensities are increased at higher ambient noise levels [24, 25]. It would be possible to include this mechanism, yet it is known to provide only provide partial compensation [25], and we would expect qualitatively similar results from the model.

Allowing ambient noise to reduce inherent information also negatively impacts on migration times. The guidance cues used by whales during their navigation are largely unknown, but listening for characteristic sound sources is certainly plausible [3, 7]: surf may allow detection of coastlines [6], while rifting of icebergs creates noise sources estimated at $\sim 245$ dB (re 1 $\mu$Pa at 1 m) [42], detectable thousands of kilometres away for the low frequency bands of baleen whale acoustics. Higher ambient noise therefore may reduce the detectability of such sources, decreasing the efficacy of target-directed motion and increasing the susceptibility of whales to ocean currents. It is also feasible that louder ambient noise reduces inherent information from other sources, such as geomagnetic field information [3], through a noise-induced reduction in processing ability due to, for example, confusion or a change in focus.

A more direct and acute response included here is noise avoidance [22, 23], where noise levels above a threshold induce movement away from the source. This can impact significantly beyond introducing deviations into migration paths. At the extreme end, migration routes may become blocked if whales are “trapped” behind a wall of noise. Noise avoidance is modelled as a ‘negative phonotaxis’ response: directed away from loud noise sources. The relative strength of noise avoidance behaviour to migration behaviour increases with the noise level; it is feasible for motion to be a balanced combination of both types of behaviour. Consequently, this acts to concentrate the population within lower noise regions, where avoidance responses are not triggered. Evidence for a noise-induced spatial redistribution of whale populations can be found in [43], following analyses into the tracks derived from detected minke whale calls before, during, and after periods of naval activity [43]. Statistical modelling further supports the hypothesis that individuals were specifically moving away from sonar-producing ships [22]. Negative phonotaxis responses could conceivably be achieved through a comparison of current noise levels with prior noise levels.

We have currently assumed a constant swimming speed. Noise avoidance, however, may also manifest in (substantial) increases to the movement speed as the individual escapes [23]. Higher speeds carry significant extra costs: energy expenditure during migration stems from both metabolism and generating propulsion. For marine animals the latter rapidly increases with speed due to the increased drag [44, 45]. Optimal migration speeds that minimise energy expenditure can be calculated for different cetacean species [46, 47]. Therefore, extending the model to include noise-modulated speed and tracking energy expenditure will allow further insight into how noise impacts on whale fitness.

More severe reactions to noise are possible beyond those included here. Numerous studies imply a causal relationship between extreme noise events and cetacean mass strandings, with close spatiotemporal correlations and biopsies indicating noise-induced physiological damage [19]. A whole spectrum of reactions is therefore plausible, from subtle to severe. Highly elevated stress could result in disorientation, potentially impacting the land avoidance mechanism that prevents beaching. Exposure to loud noises may result in transient or permanent hearing impairment, debilitating whales beyond the time of exposure [19]. These factors could be included through an additional variable, describing a whale’s current physiological state. Population heterogeneity could also be included via an age variable, that impacts both on hearing sensitivity (e.g. age-related hearing loss in older cetaceans, e.g. [48]) and navigating ability (e.g. less experienced juvenile members [3]). Incorporating these and other forms of heterogeneity, such as inherent information, or the relative weight placed on inherent or collective information, will allow us to explore whether ambient noise disproportionately impacts on different members of the group.

Each agent in our model has been taken to be an individual, effectively an assumption that whales travel singly rather than in close-knit pods. Minke whale sightings in the North Sea are usually of a single individual (e.g. [49]), but in other species/populations this may not be the case. For example, sightings of Eastern Northern Pacific gray whales are often of multiple individuals, and the reported number typically underestimates the actual pod size [50]. This extra structuring could be incorporated by allowing each agent to represent a pod of up to some number of members, raising subsequent questions on whether this provides a benefit to collective navigation. Specifically, different forms of information could be shared according to the communication between nearby pod members and the communication with distant individuals/pods.

We have made the assumption that individual whales call more frequently than they undergo reorientation events. Conservative estimates of the calling rate of Antarctic minke whales (*Balaenoptera bonarensis*), presented in [51], indicate a diel pattern with 1.93 calls per hour during daylight hours and 4.09 calls per hour during the night. A similar diel pattern of increased calls during the night was observed in the North Sea and the North-west Atlantic Ocean for northern minke whales (*Balaenoptera acutorostrata*) [39, 52]. While this is indeed more frequent than the reorientation rate in our model (once per hour, on average), it is possible that the calls are clustered together during certain behaviour, such as group foraging or when a conspecific is within close range [51]. If the calls are clustered, there may be longer periods of time between call clusters (i.e. when there are no calls), during which multiple reorientation events may occur. Alternatively, if whales rely on calls to maintain contact throughout migration, call rates may be enhanced during migratory periods. For vocally-active northern minke whales, call rates have been observed to range between 8.7 and 133.3 calls per hour [39]. It would be instructive to incorporate explicit calling behaviour in the model, so that whales respond to the most recently received signals from other whales. This will be most relevant for species that call infrequently, relative to the rate of reorientation events, as reorientation events may be based on outdated information.

Sound information has been condensed here into the intensity level, allowing us to use a simple transmission loss model [20] in which calls are heard only when the received intensity is not sufficiently below the ambient noise. A more sophisticated model could account for noises covering different frequency bands and species-specific sensitivities. However, while available for certain other cetaceans, audiograms have not yet been obtained for baleen whales [20] and the simpler intensity-based model would appear a reasonable compromise at present. Additionally, we could extend the model to account for relevant factors such as the bathymetry, ocean floor composition and reflectivity, and sound speed [34]. However, as above, the complexity of the model is chosen to be limited so that the time required to perform computational simulations remains sufficiently low.

A number of other studies have also used agent-based modelling to explore the impact of noise on whale communication space or navigation. For example, Cholewiak et al. [26] consider a model for mobile whales that swim within a region subject to shipping traffic, exploring the change in communication space for different calls across a variety of baleen populations and according to the different forms of shipping. Guarini and Coston-Guarini [32] explore the influence of bathymetry on humpback whale migrations. The work here extends the preliminary study performed in [12], and merges aspects of navigation with impacts from communication masking, while also accounting for the effects of ocean currents and bathymetry. Ocean currents can reach orders of magnitude commensurate with migration speeds and have been accounted here as a passive advection. This assumes that whales do not specifically adapt their movement with respect to an ocean current – we are not aware of any studies that suggest whales orient according to the current (rheotaxis). Nevertheless, aligning with currents is common within marine animals [53], and this could be included in future. Land avoidance was primarily included in our model to prevent beaching. However, it was also found to confer navigating benefits by funnelling whales between landmasses, improving cohesion in the process.

Our case study adopted the North Sea region due to its high level of human activity, the availability of data (noise maps [34], bathymetry [36], ocean currents [35]) and the presence of various cetaceans [54], including a seasonal aggregation of minke whales [55]. However, the modular nature of the model allows it to be adapted to other case studies: for example, noise maps are also available for Australian coastal waters [56], where humpback whales migrate along the eastern coast to breeding grounds in the Great Barrier Reef [57]. The model can also be used as a prediction tool, illustrated here by considering beneficial (lowered shipping speeds) and detrimental (increased traffic, introducing offshore construction projects) perturbations to the soundscape. It would also be possible to extend the model to consider a spectrum of potential climate change impacts, which could include altered ocean currents, changes in sound transmission due to ocean acidification or spatiotemporal changes to a target food resource. Simulating a sequence of migrations over multiple years across an age-structured whale population would allow exploration into the potential resilience of whale populations in the light of such changes.

## Materials and methods

### Study site

Our study considers the movement of a hypothetical baleen whale population along a (predominantly) south to north route across the North Sea, from a region north of the Netherlands toward the Atlantic Ocean between Scotland and the Faroe Islands. Our focus on this region is motivated by the considerable human activity in these waters, with significant levels of shipping, exploration and construction. As a by-product to this activity, there is an availability of fine-scale data (ocean noise [34], bathymetry [36] and ocean currents [35]) that form key inputs into the model. To allow fixing of certain parameters of the model (e.g. migration speeds and call source levels) we consider data for minke whales (*Balaenoptera acutorostrata*), which is the most populous species of baleen whale within the North Sea [52, 58]. The starting location coincides with previous observations of a significant seasonal aggregation near the Dogger Bank [55] region that peaks in May. However, we stress that we are not specifically modelling this species: the overall aim is to understand the potential impact of environmental factors, principally noise, on navigation. However, the model framework is flexible and can be tailored to explore specific whale migrations, given appropriate data.

### Model

The basis for the model presented here is the collective navigation model in [12]. Full details can be found in the original manuscript; however, we briefly summarise the model here. Each virtual whale is tracked according to its position (${\textbf {x}}$) and swimming orientation ($\theta$), and moves according to

1 $$\begin{aligned} \frac{d{{\textbf {x}}}}{dt} = {\textbf {v}}_{\text {active}}+{\textbf {v}}_{\text {passive}}, \end{aligned}$$

where ${\textbf {v}}_{\text {active}}$ represents the contribution to its velocity from active swimming and ${\textbf {v}}_{\text {passive}}$ is the contribution from ocean currents. Each individual undergoes active motion according to a velocity-jump random walk [59]. Individuals swim with a fixed heading ($\theta$) and speed (*s*) for an exponentially-distributed length of time before selecting a new heading, and repeating the process. The heading selection process encodes collective navigation as the selected heading reflects the inherent knowledge of a target in combination with the observed heading of other nearby individuals [12]. A detailed explanation of the model can be found in [12].

Briefly, the heading selection (i.e. choice of ${\textbf {v}}_{\text {active}}$) is a four step process. First, an individual selects a heading based on its inherent knowledge of the target. This heading is sampled from a von Mises distribution centred in the target direction with a concentration parameter corresponding to the level of inherent information available to an individual at its current location ($\kappa$). Second, the individual observes the headings of all other individuals within its perceptual range. The headings may either be explicitly signalled by the perceived individuals, or the headings may simply be observed (i.e. constructed from sequential call locations); see [31] for a detailed investigation of the impact of this choice. We do not impose an additional error term on the observed headings at this time. This remains an interesting possible model extension, where the error may depend on, for example, the background noise or the call frequency. Third, we calculate estimates for the location and concentration parameters of a von Mises distribution that would give rise to the set of headings, based on the resultant vector obtained from a weighted sum of the individual’s heading and the observed headings, according the process detailed in [12]. Finally, ${\textbf {v}}_{\text {active}}$ is sampled from the von Mises distribution with the estimated location and concentration parameters. Crucially, navigational uncertainty is captured by the spread in the set of observed headings. This previously-proposed model effectively demonstrates the benefit of collective navigation, particularly in regions of poor navigation information. However, certain model features necessary for describing long-distance whale migration are not present in the previous model. We extend the model to incorporate the influence of ocean currents, dynamic noise pollution and signal detection, noise avoidance, and land avoidance.

### Ocean currents

Ocean currents can benefit (or hinder) migration simply via passive transport according to whether the target direction is aligned (or opposed) with the dominant current direction. In certain regions, current velocities can reach speeds comparable to average migration speeds, and hence form a non-negligible contribution to motion that is incorporated through the passive transport contribution of (1). The ocean current data used here is obtained from the HYCOM model [35], which provides day-to-day currents at a spatial resolution of 0.04^∘^ latitude and 0.08^∘^ longitude. We only use the surface depth layer of this data: while baleen whales do make occasional deeper dives, we presume that while migrating they remain close to the surface, alternating between swimming and surfacing to breathe.

### Ocean noise

Ocean noise is a product of both natural processes, such as wind and rain, and anthropogenic activity, such as shipping and resource exploration, each varying spatially and temporally [17]. Shipping activity is concentrated along commercial shipping lanes, while rain occurs in transient bands. Heightened awareness of the importance of the ocean soundscape has led to increased acoustic monitoring [60] and the generation and validation of ‘soundmaps’ [34, 61]. These maps require significant computational overhead and, consequently, we utilise the data from these previously-published studies rather than explicitly modelling spatial noise distributions. Specifically, we use data generated in [34], which provides validated estimates for the noise levels across the North Sea that arise from shipping traffic and wind at the ocean surface. Separating this data according to the distinct noise sources subsequently allows us to consider both pristine (wind noise only) and current (both shipping and wind noise) soundscapes. This data also informs the parameterisation of synthetic noise maps (i.e. noise maps based on simulated traffic) based on a simplified sound transmission model. Specifically, the received level RL (dB) at a distance *r* (m) from a sound with source level SL (dB), can be modelled by

2 $$\begin{aligned} \text {RL} = \text {SL} - \gamma \log _{10} r \end{aligned}$$

where $\gamma \log _{10} r$ describes the transmission loss [20]. The coefficient $\gamma$ is bounded below by 10, corresponding to shallow water in which the spreading is effectively cylindrical, and above by 20, for deep water in which sound propagates in all directions (spherical). Here we use $\gamma = 17.8$ [62]. This is a considerable simplification; however, this makes it feasible to construct and analyse multiple synthetic noise maps, which would not be the case for detailed sound transmission models (which require the solution of partial differential equations across the ocean, accounting for accurate bathymetric data) [34]. It is plausible that baleen whales are more sensitive to specific noise bands, particularly lower frequency bands [63], and this remains a model extension of interest, if a balance can be found between fidelity of sound transmission physics and computational complexity.

### Communication range

We explicitly model potential communication masking [20], in which the call produced by a whale may be masked by the ambient noise. Specifically, we assume that a whale emits a call at 178 dB (re 1 $\mu$Pa at 1 m), consistent with median estimates of minke whale pulse trains [64], and that the transmission of this sound follows (2). This call can be detected by another individual if the signal-to-noise ratio (SNR) of the signal and the ambient noise at the location is above a theshold value

3 $$\begin{aligned} \text {SNR} = \text {RL} - N({\textbf {x}},t), \end{aligned}$$

where $N({\textbf {x}},t)$ is the ambient noise at location ${\textbf {x}}$ and time *t*. We do not make any specific assumptions about the timing of calls; only that the whales call more frequently than they undergo reorientation events, so that any information available to reorienting whales is an accurate reflection of the headings of other whales. As above, this is a considerable simplification of the complex nature of real world whale communication, where calling, transmission and detection will be frequency and orientation dependent. Plausible SNR are not restricted to positive values; for example, negative values are observed for human conversation in noisy environments [65].

### Noise avoidance

Loud noise sources have been observed to induce escape or avoidance behaviour [66]. For example, see [22, 23] with specific reference to minke whale noise avoidance responses to sonar. To include these responses into the model, we impose a mechanism where individuals reorient and move away from regions of high noise (negative phonotaxis). The strength of this behaviour is given by a sigmoidal function such that (i) below a threshold noise level there is essentially zero noise avoidance, (ii) for intermediate noise levels individuals balance noise avoidance against navigation, (iii) above a certain threshold, navigation behaviour is neglected and motion is dictated solely via noise avoidance. This latter could be viewed as essentially a stressed or panic response, where the noise overrides other factors [19]. It is likely that whales respond to different type of noise sources in different ways. One limitation of our choice of sound transmission model is that it is not straightforward to incorporate such behaviour; however, this remains an interesting avenue for a future model extension.

Here we calculate the proportion of motion that is driven by noise avoidance $w_{na}(N({\textbf {x}},t))$, where the $N({\textbf {x}},t)$ is the noise level at location ${\textbf {x}}$ and time *t*. The remaining proportion of motion (i.e. $1-w_{na}(N({\textbf {x}},t))$) is motion corresponding to regular migration behaviour. We calculate the weighting via

$$\begin{aligned} w_{na}(N({\textbf {x}},t)) = 0.5 + 0.5\tanh \Big (\frac{1}{N_s}(N({\textbf {x}},t)-N_{\text {threshold}})\Big ), \end{aligned}$$

where $N_{\text {threshold}}$ is a threshold parameter that represents the noise level at which there is an equal weighting between noise avoidance and migration and $N_s$ is the transition rate parameter that dictates the rate of transition between regular and noise avoidance behaviour. Here $N_{\text {threshold}} = 120$ dB (re 1 $\mu$Pa at 1 m) for Figs. 2 and 4, $N_{\text {threshold}} =$ 105 dB (re 1 $\mu$Pa at 1 m) (dark blue), 110 dB (re 1 $\mu$Pa at 1 m) (magenta), 115 dB (re 1 $\mu$Pa at 1 m) (orange) for Fig. 3 and $N_{\text {threshold}} = 115$ dB (re 1 $\mu$Pa at 1 m) for Figs. 5 and 6. We demonstrate the efficacy of the noise avoidance response in the Additional file 2: Fig. S2, where we place an (artificial) extreme noise source in the centre of the North Sea. We observe a clear noise avoidance response, where trajectories indicate that the whales skirt around the edge of the region of extreme noise, and then recommence normal migration behaviour.

### Land avoidance

We assume healthy whales, under normal noise levels, avoid shallow water to minimise risk of beaching. To include this behaviour, we implement a similar sigmoidal approach to land avoidance as for noise avoidance. Specifically, we use bathymetric data [36] to obtain estimates of the water depth at any given spatial coordinate; whales likely estimate distance to the ocean floor through specific downward-directed calls [7], or to the shore through listening for surf [6]. For deep water, land avoidance is essentially not considered. If the individual is in sufficiently shallow water, however, navigation is neglected and the individual prioritises motion in the direction of deepest water (we call this “bathotaxis”). At intermediate depths, there is a weighting between navigation and land avoidance.

We define a weighting $w_{la}(d({\textbf {x}}))$ that represents the proportion of motion that is in the direction of greatest water depth, given the depth at the current location $d({\textbf {x}})$. Similar to the noise avoidance response, the remaining proportion of motion (i.e. $1-w_{la}(d({\textbf {x}}))$) is motion corresponding to regular migration behaviour. We calculate the weighting via

$$\begin{aligned} w_{la}(d({\textbf {x}})) = 0.5 - 0.5\tanh \Big (0.5(d({\textbf {x}})-d_{\text {threshold}})\Big ), \end{aligned}$$

where $d_{\text {threshold}}$ is a threshold depth that represents the water depth at which there is an equal weighting between land avoidance and migration. Here we assume $d_{\text {threshold}}$ = 30 m. We note that in the present implementation of the model, land avoidance responses are balanced against noise avoidance responses (i.e. ensuring the total weights are no more than one); a failsafe mechanism is present, where any motion that would result in whales crossing onto land is aborted. However, the failsafe mechanism is not invoked in any of the scenarios we consider, i.e. there are no noise responses that drive whales onto land. We remark that this is an interesting avenue to explore, and would allow exploration into potential whale beaching events. We demonstrate the land avoidance response in Additional file 2: Fig. S3, where we observe a population of whales that are able to avoid Ireland and navigate around its coast.

### Model metrics

For the model output, we report up to 4 key statistics. All statistics are calculated across $N_{\text {repeats}} = 10$ identically-prepared realisations of the simulation. The first is the median trajectory of the population [67]. As the concept of a mean trajectory is not well defined in the presence of divergent paths (i.e. around obstacles), we follow Buchin et al. [67] and calculate the median trajectory. The median trajectory is defined by following the trajectory of an individual until it intersects with the trajectory of another individual, after which the median trajectory follows the trajectory of the other individual. The median trajectory follows this trajectory until another intersection event, after which the trajectory switches again. This approach ensures that the median trajectory always reflects a component of an actual trajectory and that the median trajectory is bound by the outermost individual trajectories [67]. We use the Matlab package “Fast Line Segment Intersection” [68] to efficiently determine intersections. We present the spread around the median trajectory by dividing space into bins and calculating the relative frequency that a whale is located in that bin. The boundary of all bins above a threshold value (0.05) is used to generate the transparent region in the figures.

The second metric is the number of whales at the target location. This is calculated by determining the average number of whales that remain in the simulation at a time and subtracting this from the initial number of whales.

The third metric is the mean distance to the target location. This is calculated by determining the centre of the whale population in an individual simulation at each time point, and calculating the Euclidean distance between the centre of the population and the target location. This is then averaged across each simulation repeat.

The fourth metric is the average number of detected whales. At each time point we calculate the number of other whales each individual whale in the population can detect. To account for the rapidly varying noise level in the ocean, we calculate the average of this across the population and across individual simulation days. This metric is then averaged across each simulation repeat.

### Data sources

As noted above, implementation of the model requires synthesis of multiple datasets: ocean current velocity data from HYCOM Global (GLBv0.08) [35], bathymetry data from EMODnet Digital Bathymetry [36], coastline data from the Global Self-consistent, Hierarchical, High-resolution, Geography Database [69], and wind-derived noise from [34]. Shipping-derived noise data is either obtained from the study [34] or from our synthetic noise model. The synthetic noise model allows incorporation of both fixed (e.g. drilling and exploration) and mobile (e.g. ships) noise sources of varying intensity, therefore permitting investigation into the influence of different shipping lanes, levels and types of traffic, or the introduction of new construction projects. We use linear interpolation between data points to evaluate each of the datasets at the location of an individual in the model. All simulations are performed in Matlab R2020b. The code used to perform the model simulations can be found at https://github.com/DrStuartJohnston/whale-migration-model.

### Parameter details

For all simulations we consider 100 whales. While we do not model a specific migration, parameters are fixed according to data for minke whales; this species is relatively abundant within the North Sea [58] and a significant subpopulation has been observed to congregate at feeding grounds at the southern end of the North Sea during spring/summer [55]. In our model the agent whales are initially distributed at random within a prespecified region, broadly compatible with the localisation of the congregation noted above and ensuring that initially the population is within communication range. For all results except Fig. 5, this is the region defined by 53.5^∘^ N to 54.5^∘^ N and 4.5^∘^ E to 5.5^∘^ E. For Fig. 5, this region is defined by 55^∘^ N to 56^∘^ N and 4.5^∘^ E to 5.5^∘^ E. The target destination of the whale population in all cases is 61^∘^ N, 5^∘^ W. Whales are considered to have arrived if they are within 50 km of the target destination. For all parameter sets we perform 10 simulation realisations. While this is not large, relative to certain other simulation studies, we are limited by the computational complexity of the simulation. We verify that the estimates of the mean and standard deviation of the migration behaviour do not change substantially with additional simulation realisations, suggesting that we are capturing the relevant behaviour with 10 simulations (Additional file 2: Fig. S11).

For all simulations we assume a constant speed of 6 km/h, within the estimated range of migration speeds for minke whales [1]. We assume a reorientation rate of, on average, once per hour. The time between reorientation events is exponentially distributed. Between reorientation events whales move with a constant velocity and the new heading selected during the reorientation process follows the procedure described in the model by Johnston and Painter [12]. Consistent with that model we select $\alpha = 0.5$ and $\beta = 0.5$, where $\alpha$ and $\beta$ each represent weighting parameters between the inherent and collective information when estimating the mean direction and uncertainty, respectively. This choice reflects the results from an exploration in [12], where an even weighting between inherent and collective information is determined to give rise to near-optimal navigation. We verify that this choice of $\alpha$ and $\beta$ is similarly appropriate for both the pristine soundscape and the current environment by repeating the study presented in Fig. 2 for different $\alpha$ and $\beta$ values. We present this result in the Additional file 2: Fig. S10. We see slightly different trends depending on the level of environmental noise, as has been observed previously [70], but $\alpha = \beta = 0.5$ remains near-optimal in both cases. It is possible that whales may adapt to different noise levels, and hence place more or less weight on the inherent information; we leave this extension for future work. We select a background level of inherent information with $\kappa = 1$; again consistent with the study in [12], this value ensures that the level of inherent navigation information can capably guide whales towards the target in the absence of collective navigation, but that collective behaviour can provide a significant boost to journey times.

Each simulation is conducted across 744 (model) hours, i.e. 31 (model) days. Note that we assume that whales do not break their migration (e.g. to rest, feed or sleep). We set the migration to begin on July 1 2010, and use the corresponding ocean current data from the HYCOM Global (GLBv0.08) model [35] with a time spacing of one day.

We assume that the source level (SL) of the whale call is 178 dB (re 1 $\mu$Pa at 1 m), consistent with previous estimates [64]. We choose a signal to noise ratio (SNR) for detection of whale calls among background noise of $\text {SNR} = -5$ dB. The minimum received level for a call to be detected is chosen to be 88 dB (re 1 $\mu$Pa at 1 m). Under our sound transmission model, this provides a (pristine) communication range of $\sim$114 km, a value chosen for its consistency with representative values of potential communication space in other studies of minke whales [25]. Note that there is an absence of audiogram data/hearing sensitivity for baleen whales [20], including minke whales, leaving uncertainty regarding their actual communication range.

Generally our model assumes the level of inherent information to be constant ($\kappa =1$). However, for Fig. 4 we consider a potential impact in which the level of inherent information is reduced in the presence of higher noise. To implement this we choose $\kappa ({\textbf {x}},t)$ as a function of the noise:

$$\begin{aligned} \kappa ({\textbf {x}},t) = \kappa _{\text {min}} + (\kappa - \kappa _{\text {min}})(0.5+0.5\tanh \Big (0.1(N({\textbf {x}},t)-N_{\text {IL}})\Big ), \end{aligned}$$

where $\kappa _{\text {min}}$ is the minimum level of inherent information (i.e. at extreme noise levels) and $N_{\text {IL}}$ is a threshold parameter that represents the noise level at which half of the inherent information (that can be lost) is lost. In Fig. 4 we use $\kappa _{\text {min}} = 0$ and $N_{\text {IL}} =$ 110 dB (re 1 $\mu$Pa at 1 m) (red), 100 dB (re 1 $\mu$Pa at 1 m) (dark blue). Note that here there is a slower transition between the two extremes (full information and no information) due to the slope parameter in the tanh function, compared to the land and noise avoidance mechanisms. This allows us to capture a range of inherent information levels across the migration.

For convenience, we present a table of all model parameters in the Additional file 2.

### Supplementary Information

**Additional file 1.** Overview, design concepts and details protocol.

**Additional file 2.** Supplementary information and additional results.

## Data Availability

The data used as inputs for the simulations can be found at https://github.com/DrStuartJohnston/whale-migration-model.

## Code availability

The code used to perform the model simulations can be found at https://github.com/DrStuartJohnston/whale-migration-model.
