# Supplementary material for: Avoidance, confusion or solitude? Modelling how noise pollution affects whale migration
Source: Mov Ecol. 2024 Feb 19;12:17. doi: 10.1186/s40462-024-00458-w (PMC10875784; doi:10.1186/s40462-024-00458-w)
Supplement: Supplementary file 1 — Additional file 1. Overview, design concepts and details protocol. [file 40462_2024_458_MOESM1_ESM.pdf]

Overview, design concepts and details protocol for "Avoidance, confusion or solitude? Modelling how noise pollution affects whale migration"

Stuart T. Johnston[1] and Kevin J. Painter[2]

[1]School of Mathematics and Statistics, The University of Melbourne, Parkville, Victoria 3010, Australia.
[2]Dipartimento Interateneo di Scienze, Progetto e Politiche del Territorio (DIST) Politecnico di Torino, Viale Pier Andrea Mattioli, Torino 39 10125, Italy.

The model description follows the ODD (Overview, Design concepts, Details) protocol for describing individual- and agent-based models [2], as updated in [3].

Purpose. The purpose of this model is to explore the interaction between noise pollution, and migrating and navigating whale populations. Specifically, we seek to investigate how different potential noise responses at the local scale manifest as different population behaviour (with respect to migration). We consider three mechanisms: noise avoidance, where whales move away from regions of noise above a specified threshold; information loss, where whales lose inherent navigation information due to noise pollution, and; loss of communication space, where the presence of noise pollution reduces the range over which whales can communicate.

Pattern. The model should provide results where a loss of either group information (due to reduced communication) or inherent information (due to a reduced ability to detect a navigation cue) at the individual scale should result in a slower rate of migration; consistent with previous models of collective navigation [4]. This is measured through the number of individuals that have arrived at a target destination over time and the average distance between the population and the target destination.

Entities. The model contains the following entities: individual agents that represent whales, and a global environment that contains information about, for example, the local noise level, ocean currents, and bathymetry. Table 1 contains the list of variables in the global environment, alongside their units and meaning. Table 2 contains the list of state variables for the agents, alongside their units and meaning.

Scales. In all cases, the model is performed on a two-dimensional continuous space simulation domain from 48 °N to 62 °N in latitude and from 16 °W to 9 °E in longitude. Note that this is an area coinciding with known distributions of baleen whale populations. To be consistent with the external environment data, we impose a discretisation such that there are 281 discrete latitude points and 301 discrete longitude points for any global environment data. Agents exist in continuous space. The model runs over the course of a single

Variable name	Variable type and units	Meaning
backgroundNoise	Real number (dB re 1m)	Ambient noise at location in time and space.
depthGrid	Real number (m)	Depth of the ocean at each location
latFlowVelocity	Real number (m/h)	Ocean current velocity in lat. direction at location in time and space.
lonFlowVelocity	Real number (m/h)	Ocean current velocity in lon. direction at location in time and space.
meanWindNoise	Real number (dB re 1m)	Mean wind noise at location in space.
shippingNoise	Real number (dB re 1m)	Ambient shipping noise at location in time and space.
navigationField	Real number	Direction of target destination at location in space.
navigationStrengthField	Real number	Level of inherent information about target destination at location in time and space.

Table 1: List of variables that are components of the global environment.

Variable name	Variable type and units	Meaning
alpha	Real number, static	Weighting of inherent and group information for heading.
avoidNoiseWeight	Real number, variable	Strength of noise avoidance response.
avoidShallowWaterWeight	Real number, variable,	Strength of shallow water avoidance response.
backgroundNoiseAtLocation	Real number, variable (dB re 1m)	Level of ambient noise at current location.
bestGuessHeading	Real number, variable	Estimate of target destination direction.
bestGuessStrength	Real number, variable	Estimate of confidence in target destination direction.
beta	Real number, static	Weighting of inherent and group information for confidence.
communicatedDirection	Real number, variable	Communicated direction of target destination.
depthAtLocation	Real number, variable (m)	Depth of water at current location.
directionAwayFromNoise	Real number, variable	Direction of maximal decrease in ambient noise at current location.
directionOfDeepestWater	Real number, variable	Direction of maximal increase in water depth at current location.
distanceFromGoal	Real number, variable (m)	Distance between the agent and the target destination.
heading	Real number, variable	Current heading of the agent.
initialPosition	Real number, static	Initial location of the agent.
latPosition	Real number, variable	Current latitude position of the agent.
lonPosition	Real number, variable	Current longitude position of the agent.
minimumHearing	Real number, static (dB re 1m)	Minimum noise signal that can be detected by an agent.
minimumNoiseOverlap	Real number, static (dB re 1m)	Signal-to-noise ratio for signal detection.
neighbours	Integer vector, variable	List of other agents detectable by the agent.
nNeighbours	Integer number, variable	Number of other agents detectable by the agent.
noiseAvoidanceLevel	Real number, static (dB re 1m)	Threshold noise level for the noise avoidance response.
position	Real vector, variable	Current (x, y) position of the agent.
potentialHeading	Real number, variable	Direction of target destination from inherent information only.
runTime	Real number, static (h)	Average time between reorientation events.
timeToUpdate	Real number, variable (h)	Time until the agent undergoes a reorientation event.
velocity	Real number, static (m/h)	Swimming speed of an agent.

Table 2: List of state variables for the agents.

month (though we note that this is trivial to change by changing *tEnd*). This month is nominally July due to the selected global environment data. Time evolves in a continuous manner. No other dimensions are represented.

Processes. The model describes the migration of a whale population that is initially located in the south of the North Sea with a target destination north of Scotland. The main components of the model are the "run" and "tumble" phases of a velocity jump random walk. The run component simply consists of ballistic motion of agents in the direction selected in the previous tumble phase combined with motion driven by immersion in ocean currents. The tumble phase is a reorientation event where inherent information, group information, land avoidance behaviour and noise avoidance behaviour are all combined to select a new heading. Each component of the reorientation event is described in the sub-models section below. In the run phase *latPosition*, *lonPosition*, *distanceFromGoal*, and *timeToUpdate* are all changed. In a reorientation event *avoidNoiseWeight*, *avoidShallowWaterWeight*, *backgroundNoiseAtLocation*, *bestGuessHeading*, *bestGuessStrength*, *communicatedDirection*, *depthAtLocation*, *directionAwayFromNoise*, *directionOfDeepestWater*, *heading*, *neighbours*, *nNeighbours* and *potentialHeading* are all updated.

Schedule. The run phase for an agent lasts for an exponentially distributed length of time, with the mean length defined by *runTime*. The tumble phase is instantaneous; a reorientation event occurs at the end of each run phase. As time is continuous and run duration is a random variable, agent reorientation is asynchronous across the population. After one reorientation event, time is simply advanced to the next reorientation event in the population.

Basic principles. The model seeks to describe the collective navigation of a whale population, where it is feasible that individual whales rely on a sophisticated synthesis of navigation cues and information from conspecifics. The broader problem can be considered as an example of the "wisdom of the crowd" phenomenon (see [1], for example), where groups are more effective at migrating than individuals. This specific model extends the previous model of Johnston and Painter [4], where confidence or uncertainty in navigation is explicitly included in the information synthesis process. Here the model is tailored to whale populations

and sound transmission in the oceans, where communication over long distances is possible under pristine ocean conditions, and the communication space is reduced under the current ocean conditions. We introduce sub-models to describe sound transmission, noise avoidance responses, land avoidance responses and inherent information loss.

Emergence. The model provides both qualitative and quantitative outcomes. It allows us to quantify the changes in summary statistics, such as the population migration rate and the percentage of successful migrations, due to changes in the global environment or parameters in the model. Moreover, clear qualitative differences in migration patterns arise in response to different types of reactions to high background noise levels. While these patterns rely on the form of the reaction (avoidance, loss of communication, loss of inherent information), these reactions are based on clear physical principles (observed retreating from extreme noise sources, and sound transmission in water).

Adaption. Agents update a considerable number of state variables (see Table 2) throughout the model simulation. The ultimate decision that is made is the new heading of the agent. This depends on the current location (via the global environmental variables of the ambient noise, bathymetry, navigation field and inherent information) and the neighbours of the agent. This process is described in depth in the reorientation model. An update of *heading* necessarily involves updating *bestGuessHeading*, *bestGuessStrength*, *heading*, *potentialHeading*, *timeToUpdate* and *communicatedDirection* (other state variables are also updated; see discussion of reorientation). The state variables of *distanceToGoal* and *position* changes constantly during the run phase. The reorientation process is not deterministic: the final choice of heading is a sample from a determined probability distribution. This can be considered as an example of indirect objective seeking, as the agents do not always progress towards the target destination (i.e. the objective).

Objectives. Agents in the model have only a single object: migrate towards a target destination until they are sufficiently close to that destination, upon which migration is classified as successful. This is tracked through the *distanceToGoal* state variable, which simply calculates the Euclidean distance between the agent and the target destination.

Learning. This model does not include learning behaviour.

Prediction. The model includes implicit prediction in both noise and land avoidance behaviour. We choose to model this behaviour via taxis-type responses, where we assume the individual can estimate or detect the spatial change in noise and ocean depth, and move down or up this gradient as appropriate. Many animals are known to move via taxis-type responses to a cue and hence it is a suitable modelling choice that will approximate known behaviour in the absence of detailed observations. There is an implicit assumption that the gradient can be estimated; however, this only relies on the ability to detect a cue nonlocally (but not over a significant distance) or remember a level of a cue at a previous location, which is plausible for whales.

Sensing. Agents are able to use all of their state variables in their decision making. The updating of the state variables involved in navigation includes uncertainty. As such, while the agents are able to reliably use state variables, the process of obtaining the state variables is where the uncertainty manifests itself. For example, *bestGuessHeading*, *bestGuessStrength*, *heading*, *potentialHeading*, *timeToUpdate* and *communicatedDirection* are all updated according to uncertain information. The distance over which an agent can detect other agents is a function of the local environment and the source level of the communication of the other agents.

Interaction. Interaction between agents is indirect and solely based on communication. Agents emit calls at a specified source level, assumed to be constant across the population. The calls propagate through the ocean environment according to a logarithmic decay model. Agents can detect emitted calls if the received level of the call is within a specified signal-to-noise ratio of the local background noise. The mathematical description of the sound propagation is defined in the sub-models section below. Interactions between agents and the environment is a purely local interaction.

Stochasticity. There are four forms of stochasticity in the model. First, the inital locations of individuals (*initalPosition*) are uniformly randomly distributed within a specified area of space. Second, the duration of each run phase (*timeToUpdate*) for each individual is sampled from an exponential distribution with mean parameter *runTime*. Third, the individual's estimate of the target destination (*potentialHeading*) is sampled from a von Mises distribution centred at the target destination direction with concentration parameter that is equal to the level of inherent information. Finally, the individual's heading (*heading*) is sampled from a von Mises distribution centred at *bestGuessHeading* with concentration parameter *bestGuessStrength*.

Collectives. There are emergent collectives of agents in the model. The reorientation process is a function of the detected headings from other agents. As such, if there are agents in close proximity, the decision making process indirectly changes. However, this is not explicitly modelled via a collective entity.

Observation. We record three metrics from the simulation (noting that others could be easily recorded). First, we record the number of agents that have reached the target destination by each time point. This is recorded at 501 time points over 744 hours (one month). Second, we record the average (Euclidean) distance between the agents and the target destination. This is recorded at 501 time points over 744 hours (one month). Finally, we record the average number of detected agents. This is calculated as a daily average and hence is recorded at 31 time points over 744 hours; the reason for the coarseness in the measurement is due to the noisiness of the data.

Initialisation. The model is initialised by loading the relevant input data (see Input data section). This includes loading the appropriate suite of parameter values for a specific case study (see Input data section). Observation metrics are initialised for later use. Agents are created, the number of which is defined in the suite of parameter values. The initial locations of agents are sampled from the appropriate uniform distributions. The initial length of the run phases are sampled from the appropriate exponential distribution.

Input data. The model relies on reading in a number of input datasets. The shipping noise (*backgroundNoise*), the wind noise (*meanWindNoise*), the coastal boundary data (*coarseLandX*, *coarseLandY*, *coarseLat*, *coarseLon*), the ocean current data (*latFlowVelocity*, *lonFlowVelocity*) and the look-up table for the von Mises likelihood (*kappaCDF*) are all loaded at the start of the simulation. The *backgroundNoise* may come from validated shipping data, synthetic shipping data (see Supplementary Information) or resource extraction platform data (see Supplementary Information). A particular parameter set (the string *parameterSet*) is loaded to describe the relevant case study or example, which contains the parameters defined in Table 3. Certain parameters are universal in all case studies considered here and are hence defined at the beginning of each simulation.

Sub-models. The key processes in the model are the reorientation events, which are composed of five different sub-models.

The first sub-model is the inherent information sub-model. When *reduceInformation* has a value of 'no' then the level of inherent information about the target destination (and hence the concentration parameter in the von Mises distribution) is constant. When *reduceInformation* has a value of 'yes' then the level of inherent information about the target destination varies with space and time. To implement this we choose $\kappa(\mathbf{x}, t)$ as a function of the noise:

$$\kappa(\mathbf{x}, t) = \kappa_{\min} + (\kappa - \kappa_{\min})(0.5 + 0.5 \tanh\Big(0.1(N(\mathbf{x}, t) - N_{\mathrm{IL}})\Big),$$

where $\kappa_{\min}$ is the minimum level of inherent information (i.e. at extreme noise levels) and N_{IL} is a threshold parameter that represents the noise level at which half of the inherent information (that can be lost) is lost. Therefore, the value of *potentialHeading* for an agent relies on the interaction between an agent's location and this function.

The second sub-model is the sound transmission model. As discussed in the main manuscript, we use a simplified sound transmission model. The received level RL (dB) at a distance r (m) from a sound with source level SL (dB), can be modelled by

$$\mathrm{RL}(r) = \mathrm{SL} - \gamma \log_{10} r$$

where $\gamma \log_{10} r$ describes the transmission loss. The coefficient γ is bounded below by 10, corresponding to shallow water in which the spreading is effectively cylindrical, and above by 20, for deep water in which sound propagates in all directions (spherical). This sound transmission model allows to calculate which agents can be detected by a specific agent. If $\mathrm{RL}(r) - N(\mathbf{x}, t) > \mathrm{SNR}$, where SNR is the signal-to-noise ratio, for a pair of agents separated by Euclidean distance r, then the agents are able to detect each other's calls. The agents are therefore classed as neighbours. The heading of all neighbour agents contribute to reorientation events and these headings are classified as group information. Full details for the synthesis of group and inherent information is given in [4]. Briefly, the weighted circular mean and resultant vector length are calculated from the heading of an agent and the headings of its neighbours. The weightings are *alpha* and *beta*, respectively. The weighted circular mean gives *bestGuessHeading* while *bestGuessStrength* is obtained from the look-up table *kappaCDF* according to the process described in [4]. The value of *heading* is then sampled from a von Mises distribution with location and concentration parameters *bestGuessHeading* and *bestGuessStrength*.

The third sub-model is the noise avoidance model. To describe noise avoidance behaviour we implement a negative phonotaxis response (i.e. motion in the direction of decreasing noise). The direction of decreasing noise is calculated using a finite difference approximation to the directional derivative from the information stored in *backgroundNoise*. The noise avoidance response is implemented in a weighted manner, where at low noise levels there is essentially no response, and at high noise levels, essentially all motion is determined by the noise avoidance response. Specifically, we calculate the proportion of motion that is driven by noise avoidance $w_{na}(N(\mathbf{x}, t))$, where the $N(\mathbf{x}, t)$ is the noise level at location $\mathbf{x}$ and time t. The remaining proportion of motion (i.e. $1 - w_{na}(N(\mathbf{x}, t))$) is motion corresponding to regular migration behaviour (i.e. in the direction of *heading*). We calculate the weighting via

$$w_{na}(N(\mathbf{x}, t)) = 0.5 + 0.5 \tanh\Big(0.5(N(\mathbf{x}, t) - N_{\text{threshold}})\Big),$$

where $N_{\text{threshold}}$ is a threshold parameter that represents the noise level at which there is an equal weighting between noise avoidance and migration. This weighting is stored as *avoidNoiseWeight*.

The fourth sub-model is the land avoidance model. We consider a similar approach for land avoidance as for noise avoidance, assuming that excessively shallow water will trigger a response in which motion is in the direction of greatest water depth (bathotaxis). As before, this relies on a finite difference approximation of the directional derivative, from the information stored in *depthGrid*. Specifically, we define a weighting $w_{la}(d(\mathbf{x}))$ that represents the proportion of motion that is in the direction of greatest water depth, given the depth at the current location $d(\mathbf{x})$. Similar to the noise avoidance response, the remaining proportion of motion (i.e. $1 - w_{la}(d(\mathbf{x}))$) is motion corresponding to regular migration behaviour (i.e. in the direction of *heading*). We calculate the weighting via

$$w_{la}(d(\mathbf{x})) = 0.5 - 0.5 \tanh\Big(0.5(d(\mathbf{x}) - d_{\text{threshold}})\Big),$$

where $d_{\text{threshold}}$ is a threshold depth that represents the water depth at which there is an equal weighting between land avoidance and migration. This weighting is stored as *avoidShallowWaterWeight*.

Reorientation events therefore occur in the following way. Based on the inherent information model, an initial heading *potentialHeading* is determined. Next, based on the sound transmission model, an agent's neighbours are calculated. The headings of the neighbours are combined with *potentialHeading* to obtain a preliminary value of *heading*. To account for land avoidance and noise avoidance, *heading* is updated to be a weighted combination of the original *heading* value, and *directionOfDeepestWater* and *directionAwayFrom-Noise*, with weightings *avoidShallowWaterWeight* and *avoidNoiseWeight*.

Variable name	Variable type and units	Meaning
includeWind	String (yes/no)	Whether wind data is included as a noise source.
includeFlowField	String (yes/no)	Whether ocean currents are included.
reduceInformation	String (yes/no)	Whether inherent information is reduced by noise pollution.
nIndividualsStart	Integer	Number of agents in the simulation.
velocity	Real number (m/h)	Swimming speed of the agents.
runTime	Real number (h)	Average time between reorientation events.
tEnd	Real number (h)	Number of hours in the simulation.
alpha	Real number	Weighting of inherent and group information for heading.
beta	Real number	Weighting of inherent and group information for confidence.
callDB	Real number (dB re 1m)	Source level of agent calls.
minimumHearing	Real number (dB re 1m)	Minimum noise signal level that can be detected by an agent.
noiseAvoidanceLevel	Real number (dB re 1m)	Threshold noise level for the noise avoidance response.
depthAvoidanceLevel	Real number (m)	Threshold depth level for land avoidance response.
minimumNoiseOverlap	Real number (dB)	Signal-to-noise ratio for signal detection.
backgroundStrength	Real number	Level of inherent information available.
goalDistance	Real number (m)	The distance to the target that an agent must get within to be counted as arrived.
nRepeats	Real number	Number of simulation realisations.
projection	Integer	The mapping projection used.
latGoal	Real number	Latitude location of the target destination.
lonGoal	Real number	Longitude location of the target destination.
startLat	Real number	Average initial latitude location of agents.
startLon	Real number	Average initial longitude location of agents.
informationMidPoint	Real number (dB re 1m)	Threshold value for the loss of inherent information.
informationDecay	Real number	Scaling in tanh function for inherent information.

Table 3: Parameter values loaded during the initialisation process.

The fifth and final sub-model is a failsafe land avoidance model. Here we store information about the location of the coastline in *coarseLandX* and *coarseLandY*. If an agent is determined to have crossed a coastline onto land, the movement is aborted. This is calculated via Matlab's *inpolygon* function, where the polygons are defined by the coastline points. We use a nested approach for efficiency, first using extremely coarse coastlines stored in *cLX* and *cLY*. If an agent is in these extremely coarse polygons, then we test via less coarse polygons. Given the tortuousity of coastlines, we cannot use fully-detailed coastline data with any reasonable computational efficiency.

References

[1] E. Codling, J. Pitchford, and S. Simpson. Group navigation and the "many-wrongs principle" in models of animal movement. *Ecology*, 88(7):1864–1870, 2007.

[2] V. Grimm, U. Berger, F. Bastiansen, S. Eliassen, V. Ginot, J. Giske, J. Goss-Custard, T. Grand, S. K. Heinz, G. Huse, et al. A standard protocol for describing individual-based and agent-based models. *Ecological modelling*, 198(1-2):115–126, 2006.

[3] V. Grimm, S. F. Railsback, C. E. Vincenot, U. Berger, C. Gallagher, D. L. DeAngelis, B. Edmonds, J. Ge, J. Giske, J. Groeneveld, et al. The odd protocol for describing agent-based and other simulation models: A second update to improve clarity, replication, and structural realism. *Journal of Artificial Societies and Social Simulation*, 23(2), 2020.

[4] S. T. Johnston and K. J. Painter. Modelling collective navigation via non-local communication. *Journal of the Royal Society Interface*, 18(182):20210383, 2021.
