# Supplementary material for: Avoidance, confusion or solitude? Modelling how noise pollution affects whale migration
Source: Mov Ecol. 2024 Feb 19;12:17. doi: 10.1186/s40462-024-00458-w (PMC10875784; doi:10.1186/s40462-024-00458-w)
Supplement: Supplementary file 2 — Additional file 2. Supplementary information and additional results. [file 40462_2024_458_MOESM2_ESM.pdf]

Supplementary information for "Avoidance, confusion or solitude? Modelling how noise pollution affects whale migration"

Stuart T. Johnston[1] and Kevin J. Painter[2]

[1]School of Mathematics and Statistics, The University of Melbourne, Parkville, Victoria 3010, Australia.
[2]Dipartimento Interateneo di Scienze, Progetto e Politiche del Territorio (DIST) Politecnico di Torino, Viale Pier Andrea Mattioli, Torino 39 10125, Italy.

Model parameters

Shipping traffic model

To investigate potential changes in shipping traffic and the impact that this change may have on whale migration we develop a model of shipping traffic and sound transmission. We define shipping routes in our domain of interest via start and end points that are connected via a number of waypoints. These routes are selected to align with established shipping routes in the North Sea. For each route, we define the number of ships that will commence along that route over the course of 100 hours. We consider five classes of ship that contribute significantly to human activity in the oceans as per MacGillivray *et al.* [4]: bulk carriers, container ships, cruise ships, tanker ships and vehicle carriers. Under normal activity in our model, bulk carriers have a mean speed of 13 knots and a mean source level of 188 dB (re 1 m). Container ships have a mean speed of 19 knots and a mean source level of 191 dB (re 1 m). Cruise ships have a mean speed of 17 knots and a mean source level of 183 dB (re 1 m). Tanker ships have a mean speed of 14 knots and a mean source level of 187 dB (re 1 m). Vehicle carriers have a mean speed of 18 knots and a mean source level of 190 dB (re 1 m). In the slowdown scenario, each ship is assumed to reduce its speed to 11 knots with a corresponding reduced mean source level of 181 dB (re 1 m) (bulk carrier), 179 dB (re 1 m) (container ships), 175 db (re 1 m) (cruise ships) and 178 db (re 1 m) (vehicle carriers).

By combining the start points, end points, waypoints and ship properties it is straightforward to calculate the location of emitted noises corresponding to individual ships. We note that the model is extremely flexible and new routes and ships can be defined as necessary; it is also possible to include other stationary or mobile noise sources, e.g. drilling or naval sonar. We incorporate the ship locations and source levels into the logarithmic model of sound transmission discussed in the Methods of the manuscript, and hence we can calculate a noise map corresponding to the synthetically-generated shipping traffic. We assume that shipping patterns do not vary significantly over the short term and allow the pattern to repeat on the scale of 100 hours.

For the results in Figure 5 we generate noise maps corresponding to three different shipping scenarios. The first scenario we consider is shipping traffic that generates migratory behaviour that is similar to that seen for the current soundscape data. We choose to replicate this to ensure that there are not discrepancies between directly compared scenarios due to different choices in sound transmission models. That is, the current soundscape data relies on a partial differential equation model of sound transmission rather than the logarithmic model considered here. The second scenario we consider is a 50% increase in shipping traffic. Specifically, we maintain the same routes, but for each route we increase the number of ships by 50%. The third scenario we consider is a 50% increase in shipping traffic (relative to current) but with sound mitigation measures applied via a slowdown. The ships emit a lower level of noise but require longer to traverse a shipping route. It is of interest to determine whether this may ameliorate the impact of the

Table 1: Table of model parameters.

**Parameter**	**Parameter meaning**	**Parameter value**	**Reference**
N_{repeats}	Number of simulation realisations	10	N/A
$N_{\text{individuals}}$	Initial number of whales	100	N/A
s	Whale swimming speed (m h^{-1})	6000	[1]
μ	Reorientation rate (h^{-1})	1	N/A
t_{end}	Final simulation time (h)	744	N/A
α	Inherent information weighting (mean)	0.5	[3]
β	Inherent information weighting (concentration)	0.5	[3]
κ	Background level of inherent information	1	[3]
SL	Source level of whale call (dB re 1 μPa at 1 m)	178	[6]
RL_{min}	Minimum detectable call (dB re 1 μPa at 1 m)	88	N/A
N_s	Noise avoidance transition parameter	0.5	N/A
$N_{\text{threshold}}$	Noise avoidance threshold (dB re 1 μPa at 1 m) (Fig. 2,4)	120	N/A
$N_{\text{threshold}}$	Noise avoidance threshold (dB re 1 μPa at 1 m) (Fig. 3)	105, 110, 115	N/A
$N_{\text{threshold}}$	Noise avoidance threshold (dB re 1 μPa at 1 m) (Fig. 5, 6)	115	N/A
$d_{\text{threshold}}$	Land avoidance threshold (m)	30	N/A
SNR	Signal-to-noise ratio for detectable calls (dB re 1 μPa at 1 m)	-5	N/A
γ	Logarithmic sound transmission decay parameter	17.8	[5]
d_{target}	Distance to target to be counted as arrived (m)	50,000	N/A
$\mathbf{x}_{\text{target}}$	Target location	61° N, 5° W	N/A
$\mathbf{x}_{\text{initial}}$	Initial location (Fig. 2-4)	53.5° N, 4.5° E	N/A
$\mathbf{x}_{\text{initial}}$	Initial location (Fig. 5)	55° N, 4.5° E	N/A
$\mathbf{x}_{\text{spread}}$	Spread in initial location	1° N, 1° E	N/A
$\text{SL}_{\text{construction}}$	Source level of construction activity (dB re 1 μPa at 1 m)	210	[2]

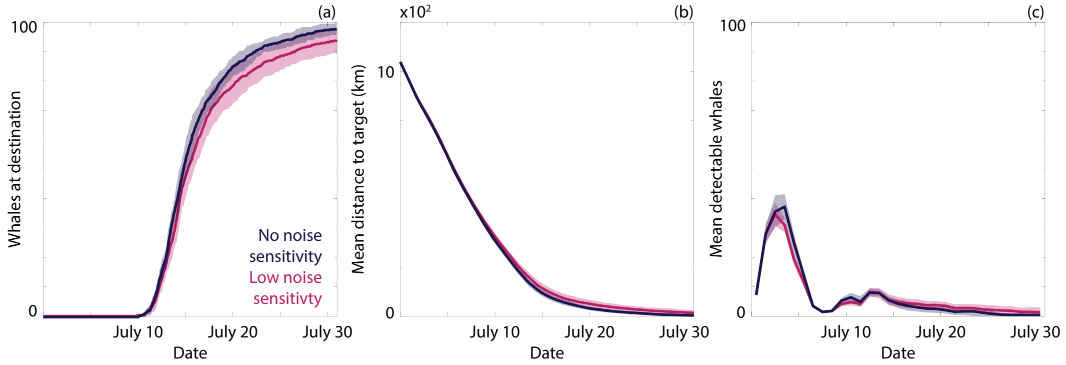

Figure 1: **Comparison of navigation with different sensitivities to noise that induce an avoidance response**. (a) The number of whales that have arrived at the target destination for no noise sensitivity (dark blue) and low noise sensitivity (magenta). (b) The mean distance of the population from the target for no noise sensitivity (dark blue) and low noise sensitivity (magenta). (c) The mean number of detectable whale calls (averaged daily) for no noise sensitivity (dark blue) and low noise sensitivity (magenta). The lines and ribbons correspond to the mean ± one standard deviation over 10 simulations.

presence of additional ships. We consider 13 shipping routes (each of which can be traversed forward or backward, for a total of 26 routes). The precise start points, end points and waypoints can be found in the code repository; we also present a representative image that contains this information in Fig. 5 (main manuscript).

Construction model

To investigate the impact of potential future construction activity on whale migration we examine the introduction of an intermittent noise source. This noise source is considered to be a representation of the noise emitted during pile driving activity in the construction of, say, an oil rig or an offshore wind farm. The source level of the construction activity, while active, is chosen to be 210 dB (re 1 m), consistent with observations [2]. The location of the construction activity is chosen to be at 56.35 °N, 4.25 °E, though we comment this location is for illustrative purposes only, rather than reflecting any specific construction plans. We consider a range of scenarios involving different levels of activity. These include patterns of work of (i) no activity; (ii) 5 hours of activity, followed by 19 hours of inactivity; (iii) 8 hours of activity, followed by 16 hours of inactivity, and; (iv) 12 hours of activity, followed by 12 hours of inactivity. We examine the situation where this construction activity is occurring in either a pristine soundscape (Fig. 5) or the current soundscape (Fig. 4). We also examine the impact of redistributing the times of activity while maintaining the total activity. Here we consider (i) 6 hours of activity, followed by 18 hours of inactivity, every day; (ii) 9 hours of activity, followed by 15 hours of inactivity, for two days followed by a day of no activity, and; (iii) 12 hours of activity, followed by 12 hours of inactivity, followed by a day of no activity. The results are presented in Figure 5. Note that there is, on average, 6 hours of construction activity each day. In each simulation we randomly select which day of construction activity corresponds to the first day in the simulation. We observe extremely similar results for each construction schedule, which suggests that the total amount of construction activity has a more significant impact than the distribution of the activities.

Sensitivity analysis

We conduct a sensitivity analysis to examine how the median arrival time of the population varies with changes in the model parameters, with a particular focus on model parameters that are not readily able to be estimated. We first consider changes to the level of inherent information available to individuals and present the median arrival time for the population in the pristine and current soundscapes in Figure 6. We observe a monotonic, but plateauing, decrease in median arrival time with an increase in the inherent

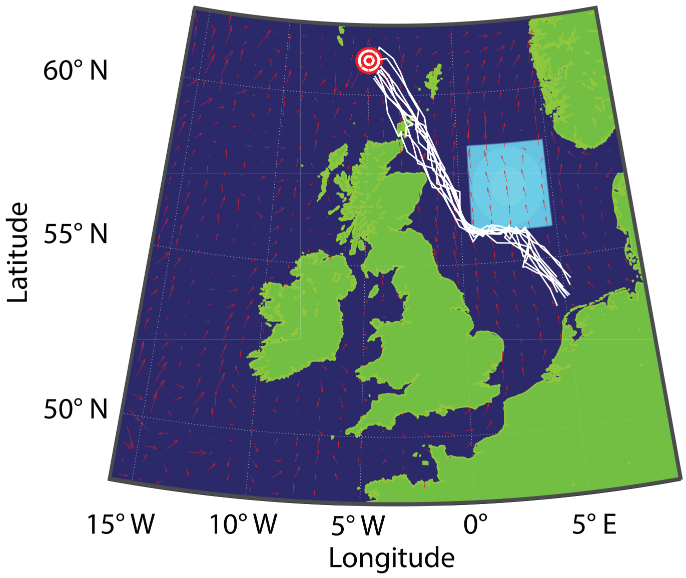

Figure 2: **Demonstration of noise avoidance**. Representative trajectories of whales avoiding an extreme noise source (cyan) located in the middle of a migration path.

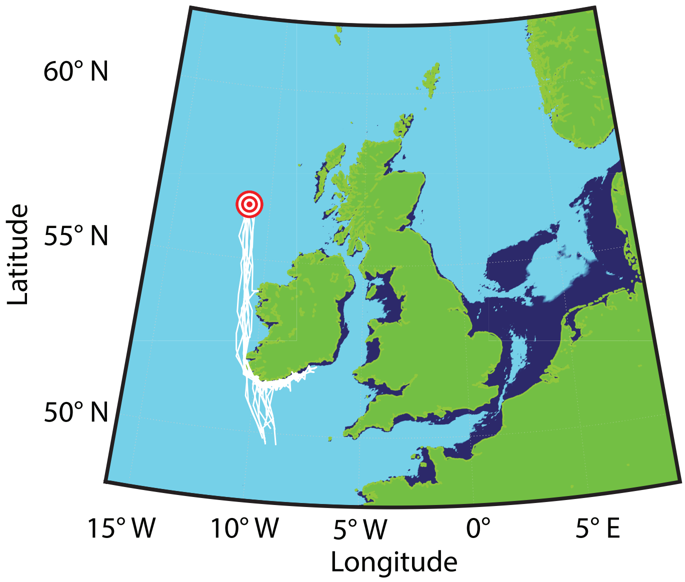

Figure 3: **Demonstration of land avoidance**. Representative trajectories of whales avoiding land located in the middle of a migration path. Cyan regions correspond to ocean depths of greater than 40m, dark blue regions correspond to ocean depths of less than 40m.

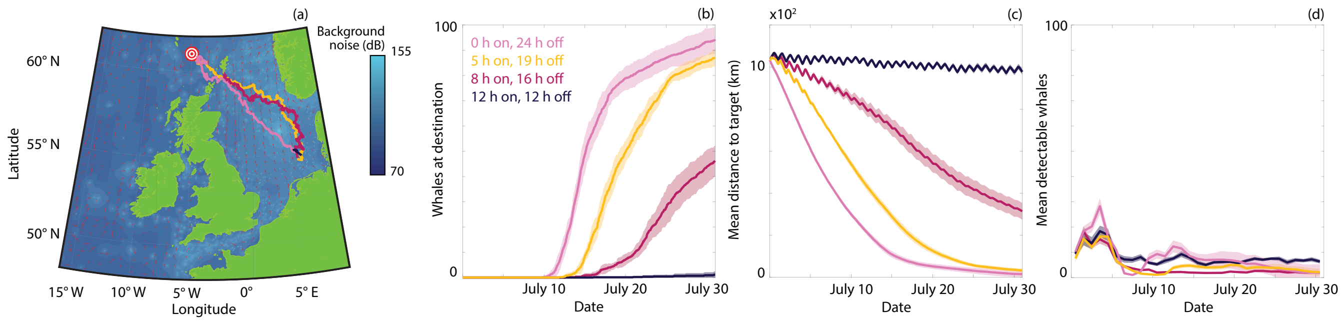

Figure 4: **Comparison of navigation under different levels of construction activity in the current soundscape**. (a) Noise map during active construction at 56.35 °N, 4.25 °E with median trajectories for 0 hours (pink), 5 hours (orange), 8 hours (magenta) or 12 hours (dark blue) of construction activity per day. (b) The number of whales that have arrived at the target destination with 0 hours (pink), 5 hours (orange), 8 hours (magenta) or 12 hours (dark blue) of construction activity per day. (c) The mean distance of the population from the target for 0 hours (pink), 5 hours (orange), 8 hours (magenta) or 12 hours (dark blue) of construction activity per day. (d) The mean number of detectable whale calls (averaged daily) for 0 hours (pink), 5 hours (orange), 8 hours (magenta) or 12 hours (dark blue) of construction activity per day. The lines and ribbons correspond to the mean $\pm$ one standard deviation over 10 simulations. In all simulations there are 100 whales.

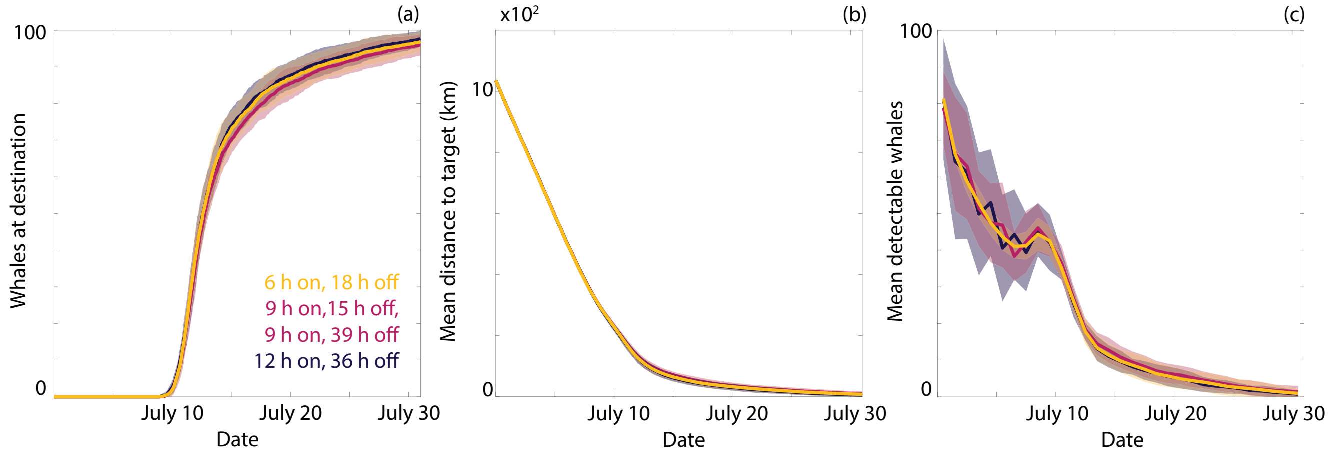

Figure 5: **Comparison of navigation under different schedules for construction activity in the pristine soundscape**. (a) The number of whales that have arrived at the target destination with 6 hours daily (orange), 9 hours for two days, followed by an off day (magenta), 12 hours for one day, followed by an off day (dark blue) of construction activity. (c) The mean distance of the population from the target with 6 hours daily (orange), 9 hours for two days, followed by an off day (magenta), 12 hours for one day, followed by an off day (dark blue) of construction activity. (d) The mean number of detectable whale calls (averaged daily) with 6 hours daily (orange), 9 hours for two days, followed by an off day (magenta), 12 hours for one day, followed by an off day (dark blue) of construction activity. The lines and ribbons correspond to the mean $\pm$ one standard deviation over 30 simulations. In all simulations there are 100 whales.

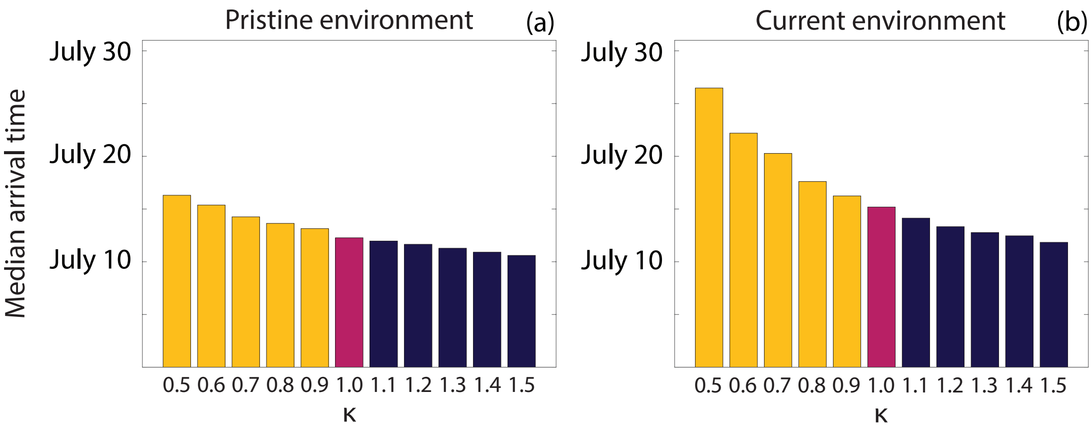

Figure 6: **Sensitivity of migration timing to inherent information**. Median arrival time for a population of 100 whales for different levels of inherent information in (a) the pristine soundscape and (b) the current soundscape. Results are the mean of 10 identically-prepared realisations of the simulation. Magenta indicates the parameter value used in the main manuscript, orange indicates parameter values that might be expected to delay arrival times, dark blue indicates parameter values that might be expected to expedite arrival times.

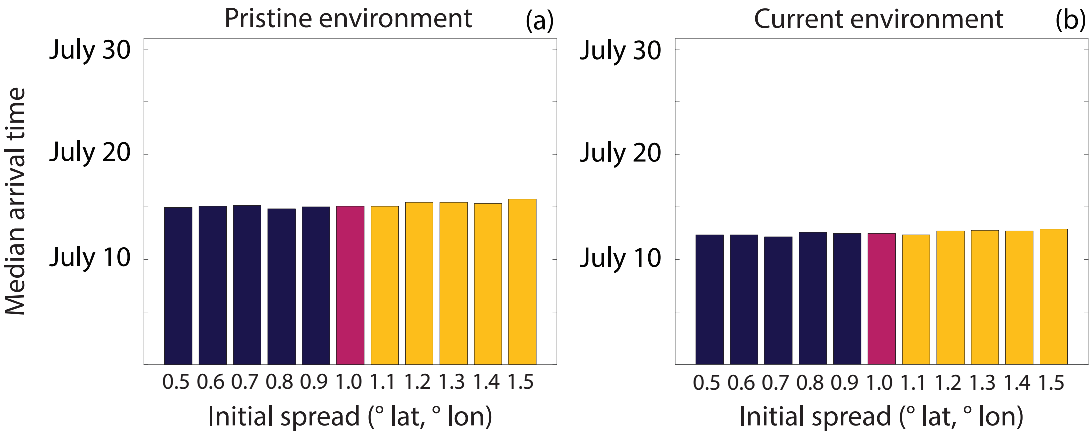

Figure 7: **Sensitivity of migration timing to the initial spread of the population**. Median arrival time for a population of 100 whales for different levels of inherent information in (a) the pristine soundscape and (b) the current soundscape. Results are the mean of 10 identically-prepared realisations of the simulation. Magenta indicates the parameter value used in the main manuscript, orange indicates parameter values that might be expected to delay arrival times, dark blue indicates parameter values that might be expected to expedite arrival times.

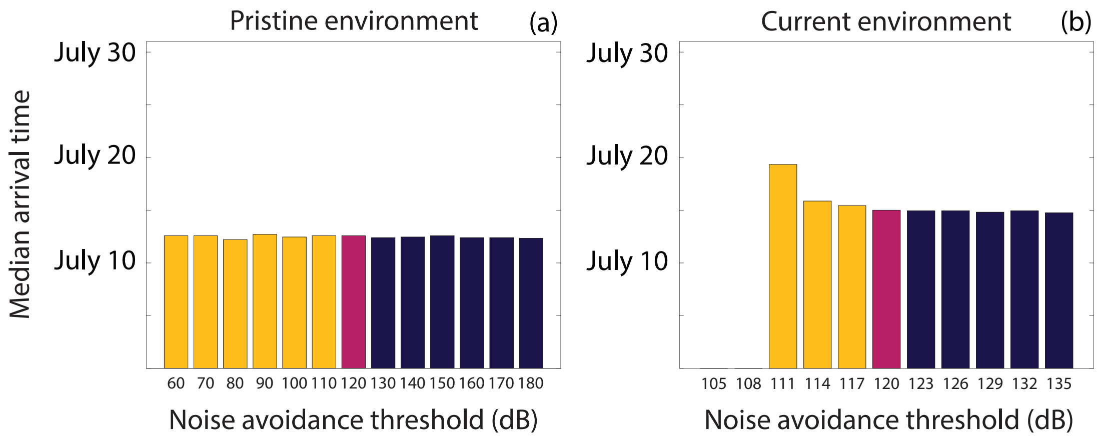

Figure 8: **Sensitivity of migration timing to the noise avoidance threshold**. Median arrival time for a population of 100 whales for different levels of inherent information with (a) low sensitivity to noise and (b) intermediate sensitivity to noise. Results are the mean of 10 identically-prepared realisations of the simulation. Magenta indicates the parameter value used in the main manuscript, orange indicates parameter values that might be expected to delay arrival times, dark blue indicates parameter values that might be expected to expedite arrival times.

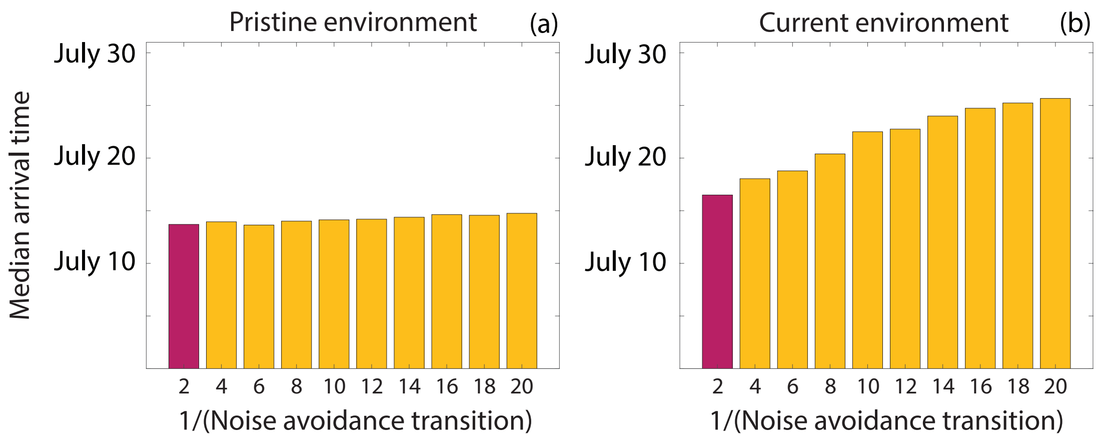

Figure 9: **Sensitivity of migration timing to the noise avoidance transition parameter**. Median arrival time for a population of 100 whales for different levels of inherent information in (a) the pristine soundscape and (b) the current soundscape. Results are the mean of 10 identically-prepared realisations of the simulation. Magenta indicates the parameter value used in the main manuscript and orange indicates parameter values that might be expected to delay arrival times.

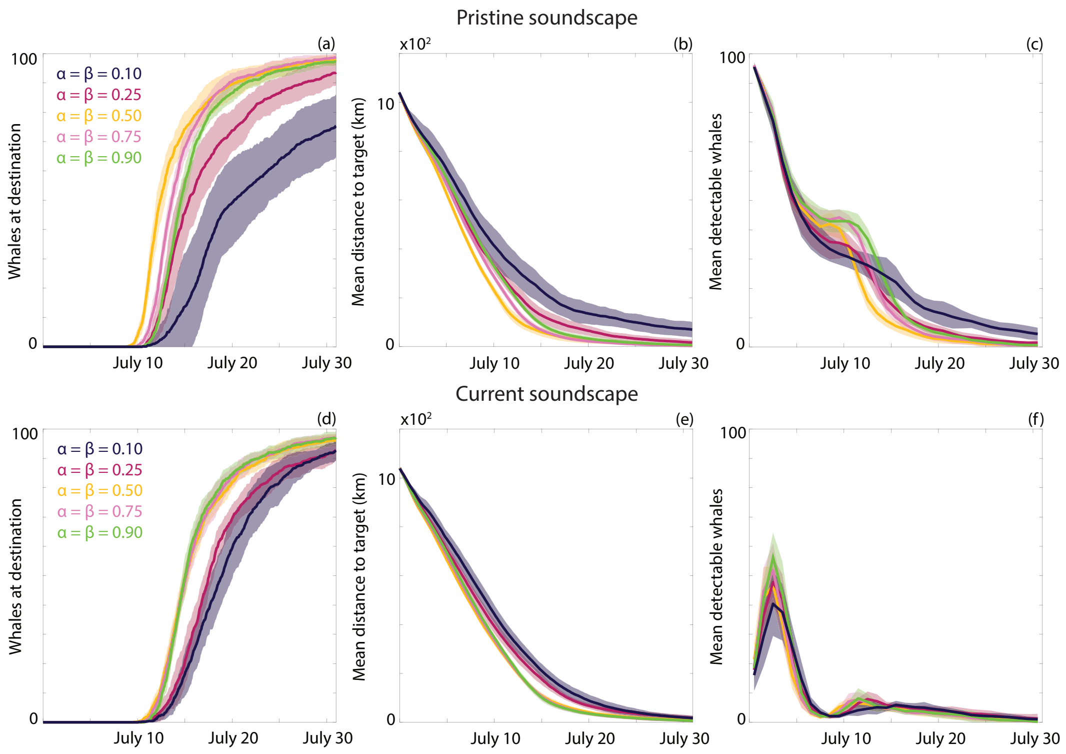

Figure 10: **Comparison of navigation under different weights of inherent and collective information in both (a)-(c) the pristine and (d)-(f) the current soundscape**. (a),(d) The number of whales that have arrived at the target destination. (b),(e) The mean distance of the population from the target. (c),(f) The mean number of detectable whales (averaged daily). Results are presented for $\alpha = \beta = 0.1$ (dark blue), $\alpha = \beta = 0.25$ (magenta), $\alpha = \beta = 0.5$ (orange), $\alpha = \beta = 0.75$ (pink) and $\alpha = \beta = 0.9$ (green). The lines and ribbons correspond to the mean $\pm$ one standard deviation over 10 simulations. In all simulations there are 100 whales.

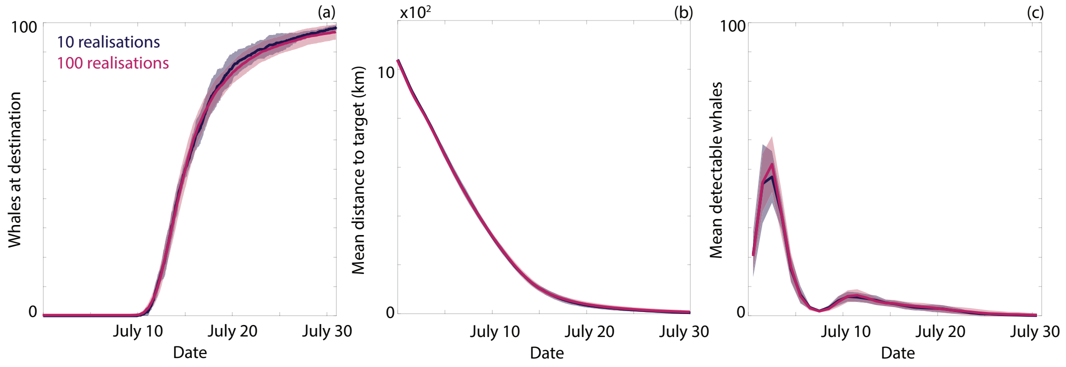

Figure 11: **Comparison of navigation for different numbers of simulation realisations**. (a) The number of whales that have arrived at the target destination averaged over 10 realisations (dark blue) and 100 realisations (magenta). (b) The mean distance of the population from the target averaged over 10 realisations (dark blue) and 100 realisations (magenta). (c) The mean number of detectable whales (averaged daily) averaged 10 realisations (dark blue) and 100 realisations (magenta). The lines and ribbons correspond to the mean $\pm$ one standard deviation. In all simulations there are 100 whales.

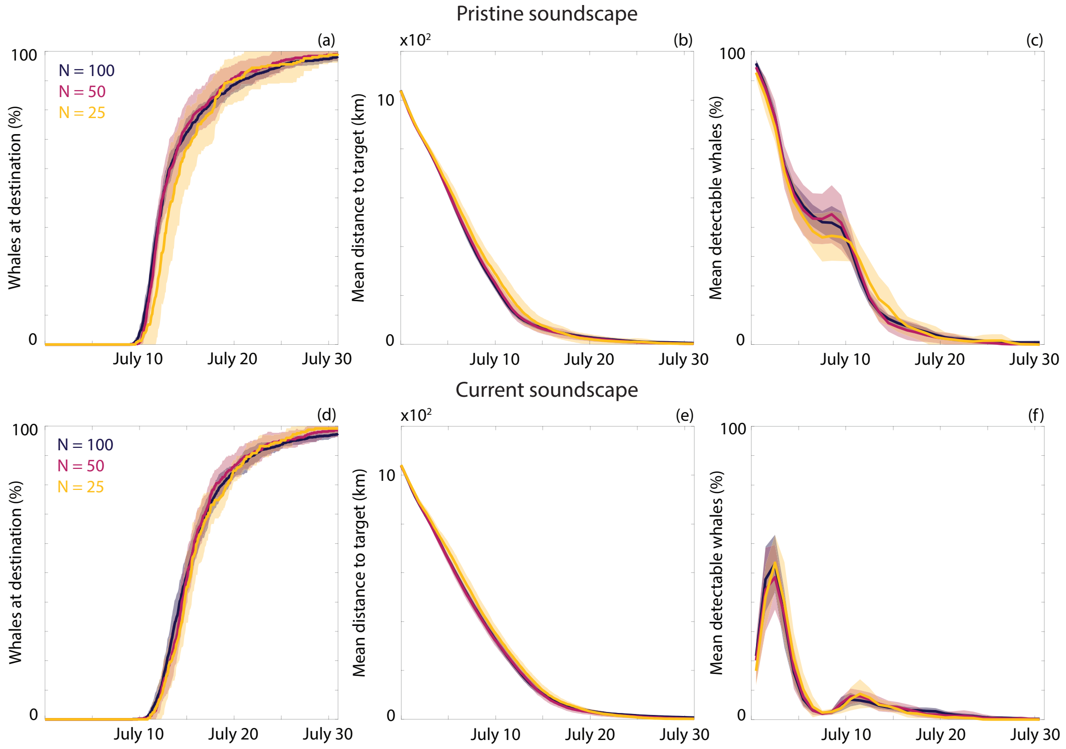

Figure 12: **Comparison of navigation for differently sized whale populations**. (a) The percentage of whales that have arrived at the target destination for a population of size 25 (orange), 50 (magenta) or 100 (dark blue). (b) The mean distance of the population from the target for a population of size 25 (orange), 50 (magenta) or 100 (dark blue). (c) The mean number of detectable whales (averaged daily) for a population of size 25 (orange), 50 (magenta) or 100 (dark blue). The lines and ribbons correspond to the mean $\pm$ one standard deviation over 10 simulations.

information. This is consistent with the results presented in Johnston and Painter [3]. Further, the results indicate that the population is not at risk at migration failure due to the ocean currents overcoming effective navigation ability for the parameter values selected in the main manuscript.

We next consider changes to the initial spacing of the population and present the results in Figure 7. We observe that the median arrival time is insensitive to the initial spread of the population. This is partially due to the fact that the bathymetry can induce aggregation through avoidance of regions of shallow water.

We now consider the influence of the noise avoidance threshold parameter. We perform simulations in the pristine and current soundscapes and present the results in Figure 8. Unsurprisingly, for the pristine soundscape, the median arrival time is insensitive to a wide range of noise avoidance threshold parameter values as there are very few regions where noise avoidance is relevant for the parameters considered. In contrast, for the current soundscape, the median arrival time is highly sensitive. For noise avoidance threshold parameter values that are well above the background noise, we see no change to the median arrival time. However, there is a rapid transition between a consistent median arrival time and a complete failure of migration. This highlights the need to understand the response of whales to extreme noise sources.

We consider how the rate of transition between noise avoidance behaviour and regular migration behaviour impacts the median arrival time. We present the results in Figure 9. A decrease in the rate of transition implies that there is weak, but increased, noise avoidance behaviour at lower noise levels, while there is reduced noise avoidance behaviour at higher noise levels (relative to the original rate of transition). We observe that as the transition becomes slower, the median arrival time increases. This is because it is more common for there to be a non-negligible component of behaviour that is dictated by noise avoidance, compared to a sharp transition. However, experimental observations indicate that this type of slow transition is less likely as there appears to be a threshold noise level below which cetaceans do not avoid the noise source.

We investigate how the weighting of inherent and collective information for estimation of the von Mises distribution parameters (α and β). Similar to the results in Johnston and Painter [3], we see that in environments that allow considerable communication between individuals (Figures 10(a)-(c)) that an equal weighting between inherent and collective information is approximately optimal. We observe that weights that represent near-complete reliance on either inherent or collective information correspond to migration that is less effective. For environments where communication is limited (Figures 10(d)-(f)), we see that an equal weighting is similarly effective, with weights that represent heavier reliance on inherent information also corresponding to effective migration. This is consistent with the results presented in [3].

In Figure 11, we verify that 10 simulation realisations provides a sufficiently accurate estimate of both the mean migration behaviour and the measure of variation between simulations. We see that the lines and ribbons overlap and are extremely similar for 10 and 100 realisations, noting a small amount of noise is present in the case with 10 realisations.

In Figure 12, we examine how the migration behaviour is influenced by the number of individuals included in the simulation. As might be expected, we see that there is a reduction in migration speed for smaller populations, as there is less collective migration. This is more pronounced in the pristine soundscape (Figures 12(a)-(c)), as there is limited communication in the current soundscape that is lost due to the smaller number of individuals (Figures 12(d)-(f)). Indeed, aside from the early aggregation the current soundscape, there are few detectable whales so we observe similar behaviour for different numbers of individuals. In contrast, there is communication throughout the migration in the pristine soundscape, and hence we see additional benefit for more individuals, consistent with [3].

References

[1] T. W. Horton, D. M. Palacios, K. M. Stafford, and A. N. Zerbini. Baleen whale migration. In *Ethology and Behavioral Ecology of Mysticetes*, pages 71–104. Springer, Cham, Switzerland, 2022.

[2] L.-F. Huang, X.-M. Xu, L.-L. Yang, S.-Q. Huang, X.-H. Zhang, and Y.-L. Zhou. Underwater noise characteristics of offshore exploratory drilling and its impact on marine mammals. *Frontiers in Marine Science*, 10:67, 2023.

[3] S. T. Johnston and K. J. Painter. Modelling collective navigation via non-local communication. *Journal of the Royal Society Interface*, 18(182):20210383, 2021.

[4] A. O. MacGillivray, Z. Li, D. E. Hannay, K. B. Trounce, and O. M. Robinson. Slowing deep-sea commercial vessels reduces underwater radiated noise. *The Journal of the Acoustical Society of America*, 146(1):340–351, 2019.

[5] A. Širović, J. A. Hildebrand, and S. M. Wiggins. Blue and fin whale call source levels and propagation range in the Southern Ocean. *The Journal of the Acoustical Society of America*, 122(2):1208–1215, 2007.

[6] D. Wang, W. Huang, H. Garcia, and P. Ratilal. Vocalization source level distributions and pulse compression gains of diverse baleen whale species in the Gulf of Maine. *Remote Sensing*, 8(11):881, 2016.
